# Landscape and Clinical Significance of Immune Checkpoint in Cutaneous Melanoma

**DOI:** 10.3389/fimmu.2021.756282

**Published:** 2021-12-24

**Authors:** Rui Mao, Fan Yang, Tongtong Zhang, Ji Li

**Affiliations:** ^1^ Department of Dermatology, Xiangya Hospital, Central South University, Changsha, China; ^2^ Emergency Department, Peking University Third Hospital, Peking University School of Medicine, Beijing, China; ^3^ The Center of Gastrointestinal and Minimally Invasive Surgery, The Third People’s Hospital of Chengdu, Chengdu, China; ^4^ Medical Research Center, The Third People’s Hospital of Chengdu, The Affiliated Hospital of Southwest Jiaotong University, The Second Chengdu Hospital Affiliated to Chongqing Medical University, Chengdu, China; ^5^ Hunan Key Laboratory of Aging Biology, Xiangya Hospital, Central South University, Changsha, China

**Keywords:** skin cutaneous melanoma, immune checkpoint, prognosis, nomogram, immunotherapy

## Abstract

**Background:**

The incidence of cutaneous melanoma (CM) is increasing, and its prognosis is not optimistic. Although immune checkpoint (ICP) inhibitors are effective in the treatment of CM patients, they are not effective for all CM patients. There is an urgent need for a marker to predict both the prognosis and the immunotherapy effect in patients with CM.

**Approaches:**

Two groups of patients with greatly different prognosis and response to immunotherapy were identified by unwatched cluster exploration of TCGA on the basis of 34 ICPs. The prognosis and immunotherapy effect of CM were predicted by developing a precise and given signature on the basis of ICPs, and a multivariate Cox risk regression model was established from the TCGA cohort consisting of 454 CM samples. The model was validated in 210 and 231 samples in the test and verification cohorts, respectively.

**Results:**

The prognosis in clinical subgroups was predicted by the classification system. High-risk patients had poorer responses to chemotherapy and immunotherapy. Finally, the signature was recognized as an independent prognostic factor. Based on checkpoint-based signature (ICPBS) and clinical characteristics, we constructed a nomogram for the prognosis in patients with CM, which was superior to ICPBS in efficacy than ICPBS alone.

**Conclusion:**

As a useful prognostic tool to further improve cancer immunotherapy, the signature can accurately predict recurrence and overall survival among patients with CM.

## Introduction

Based on the latest cancer statistics in 2020 (1), cutaneous melanoma (CM) is the fifth most common cancer among men and the sixth most common cancer among women worldwide. The incidence of CM has been rising steadily (2, 3). Although some patients with metastatic melanoma greatly benefit from immunotherapy, others either become drug-resistant or show no response at all (4, 5). These clinical challenges require finding new drug targets and drugs that can benefit patients who are intrinsically resistant or resistant to targeted therapy and immunotherapy (6).

There has been research on immunotherapy for various solid tumors, including CM (7). CM protected from disruption by T cells (8) shows a common feature of the increased expression of inhibitory checkpoint proteins [such as programmed cell death 1 ligand 1 (PDL1), PDL2, programmed cell death 1 (PD1), and cytotoxic T lymphocyte protein 4 (CTLA4)]. Immune checkpoint (ICP) therapy enhances antitumor immune responses (9, 10) by targeting these usually inhibitory signals expressed on T and tumor cells. Except checkpoint proteins, co-inhibitory ligands including B7-H3 (also referred to as CD276) and B7X (also referred to as B7-H4) also show an increased expression level in many solid cancers such as CM. The overexpression of other hidden immune checkpoint proteins, including HHLA2 (11), TMIGD2, TIM3 (12), and LAG3, is found in several tumors where immune suppression (13, 14) is mediated.

As a significant factor, the immune response in the cancer microenvironment determines tumor invasion, progression, and response to immunomodulators. There have been extensive studies on the density and types of tumor infiltrate immune cells as well as their cytokine and immune gene expressions as prognostic biomarkers for CM (15). Moreover, according to previous studies, there is significant value in predicting recurrence and prognosis among patients with CM (16) by applying immune-related gene or tumor-infiltrating immune cell signatures. However, further research on whether these immune checkpoint signatures can also be applied to predict the recurrence and prognosis of CM is needed.

Given that more than 50% of CM patients have poor results and poor prognosis after immunotherapy (17), better prognostic biomarkers are needed to accurately identify patients that would benefit from these treatments. In this context, it is important to study an immune checkpoint-based prognosis signature (ICPBS) among patients with CM. Through comprehensive analysis of the immune checkpoints and tumor microenvironment in CM, the clinical stratification of the risk in CM patients can be improved, and possible biotherapeutic targets can be explored. The present study examined 690 CM cases with overall survival (OS) data and 210 CM cases with distant metastasis-free survival (DMFS) and disease-specific survival (DSS) data from six separate cohorts, namely, TCGA, GSE22153, GSE22154, GSE46517, GSE54467, and GSE65904, to set up a new robust ICPBS. We also explored the clinical and pathological features as well as the immune landscape of the signature.

## Materials and approaches

### Data Collection and Preprocessing

**Figure 1:**
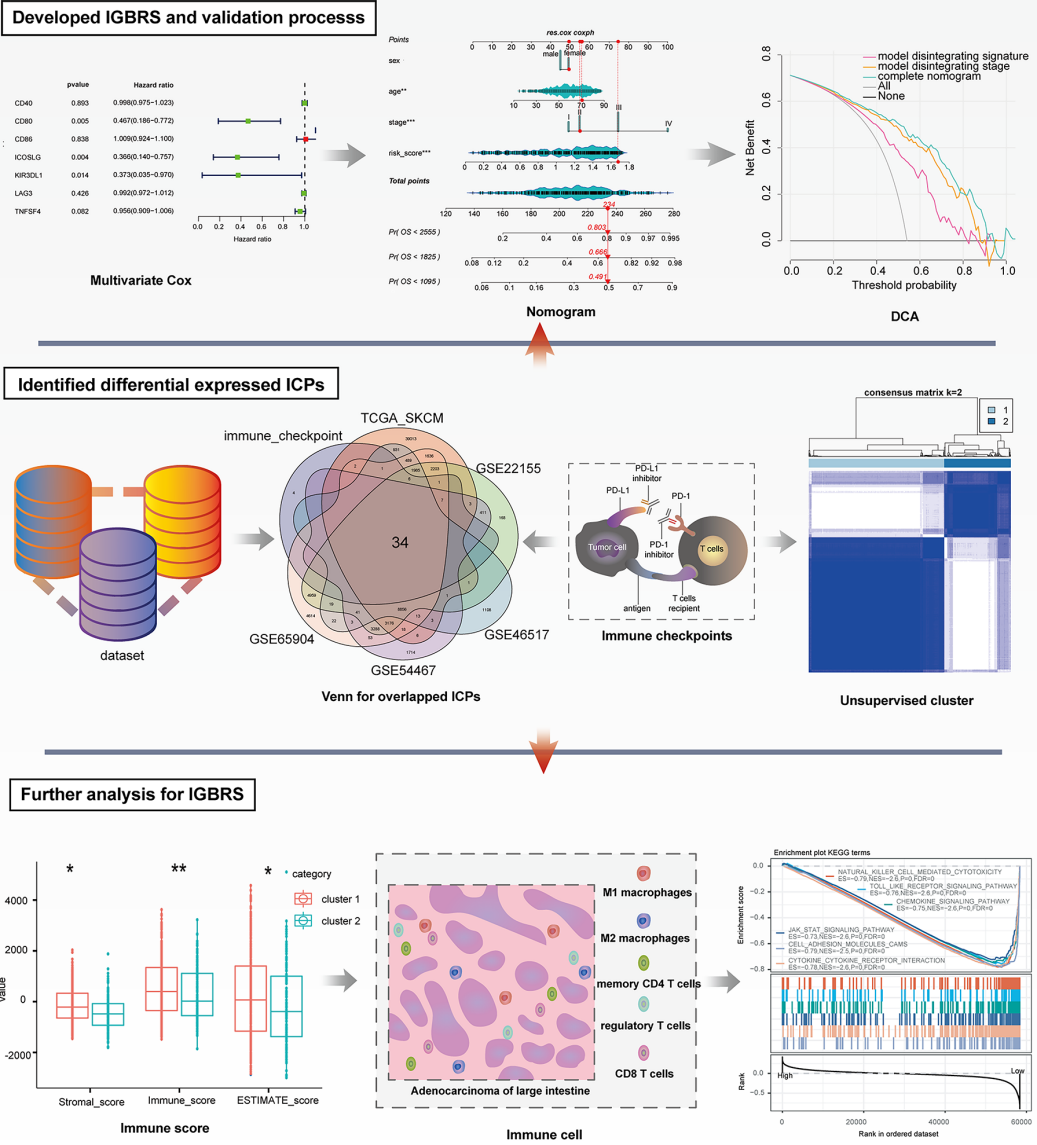
The entire analytical process of the study.

**Figure 1** shows the whole analytical process of the study.

The GEO (https://www.ncbi.nlm.nih.gov/gds/) database provided the GSE22153, GSE22154, GSE46517, GSE54467, and GSE65904 datasets. GSE54467 was downloaded with log2-transformed and quantile-normalized matrices, while GSE22153, GSE22154, GSE46517, and GSE65904 datasets had to normalize, subsequently. In principle, genes with multiple probes were expressed on the basis of the median. The mRNA expression profiles were presented in the fragments per kilobase million (FPKM) format. The information used in this research satisfied the following criteria: (1) mRNAs with nonzero expression degrees occupied 75% of all samples; and (2) the patients had accurate follow‐up times. TCGA (https://portal.gdc.cancer.gov/) provided TCGA-SKCM dataset that was used as a training cohort and included mRNA expression profiles of 454 CM specimens and the related clinical follow‐up data.

GSE65904 datasets were used as a test cohort as these datasets contained DSS (210 patients) and DMFS (150 patients) information. Moreover, the GSE22153, GSE22154, GSE46517, and GSE54467 datasets were integrated as a verification cohort as these datasets contained single OS (236 patients) information. While integrating the GSE datasets, the batch effect was removed by the combat role of the R software package “sva”. **Supplementary Table 1** shows the basic data of the datasets included in this study.

A total of 49 immune checkpoints were involved in this study, including the B7-CD28 family [CD274 (PD-L1), CTLA4, B7-H3, ICOSLG, TMIGD2, PD-L2, ICOS, PD-1, and HHLA2] (18, 19), the TNF superfamily (TNFRSF14, TNFRSF18, TNFRSF25, TNFRSF4, TNFRSF8, TNFRSF9, TNFSF14, TNFSF15, TNFSF18, TNFSF4, TNFSF9, CD40, BTLA, CD27, CD40LG, and CD70) (20), and some other immune checkpoint members (BTNL2, ADORA2A, CD160, CD200R1, CD200, CD244, CD44, CD28, CD80, CD48, CD86, FGL1, ENTPD1, HAVCR2, IDO2, KIR3DL1, IDO1, LAG3, LAIR1, NRP1, LGALS9, NCR3, and TIGIT) (21–23).

### Principal Component Analysis

Applying the R package “psych”, “reshape2”, and “factoextra”, we performed PCA on four subsets of the validation dataset based on the expression of 34 immune checkpoint molecules. The results were visualized using R package “ggplot2”.

### Unsupervised Clustering for Immune Checkpoints

Based on the expression of immune-related genes (IRGs), patients were classified by unwatched cluster exploration. The consensus clustering algorithm (24) determined the number and stability of the clusters. These steps were carried out on the basis of the ConsensuClusterPlus package (25) and repeated 1,000 times to ensure the stability of the classification (maxK = 10, reps = 1000, pItem = 0.8, pFeature = 1, and clusterAlg = “pam”). The code for unsupervised cluster analysis and the input file are available as supplementary source files.

### Analysis of the Effect of Immunotherapy

We analyzed the possible effect of immune checkpoint inhibition in patients with melanoma by Tumor Immune Dysfunction and Exclusion (26) (TIDE, http://tide.dfci.harvard.edu/). In the analysis, patients with TIDE scores greater than 0 were officially defined as nonresponders to immune checkpoint inhibitor therapy, while those with a TIDE score less than 0 were defined as respondents (26).

### Identification of Differentially Expressed Genes in Cluster 1 and Cluster 2

The difference of genes in cluster 1 and cluster 2 was analyzed by the edgeR package in R software (27). Modified *p*-values < 0.05 and |log2FC (fold variation)| > 1 (|log2FC > 1| and the modified FDR < 0.05) were set to identify significantly expressed RNAs. The ggplot2 package was used to plot a volcano map.

### Module Function Annotation

We carried out KEGG enrichment analysis and immune pathway enrichment analysis by using the ClueGO (28) plug-in in Cytoscape (version 3.8.2) and visualized it. In addition, significant GO or KEGG terms or genes were defined as those with the adjusted-*p* < 0.05 and at least three related mRNAs.

### Associations Between the Clusters and the Immunity

The immune score of every sample with R software were determined by the ESTIMATE algorithm, and the Wilcoxon test (29) was used to further compare the variations in the extent of immune cell infiltration among various cluster groups. The ratios of 22 and 10 immune cell subtypes were assessed by the CIBERSORT and MCPcounter package on the basis of the expression file, respectively (30). Further analyses adopted the samples with *p* < 0.05 in the CIBERSORT and MCPcounter exploration outcomes. The comparison of variations in immune cell subtypes among different cluster groups was made by the Mann–Whitney *U* test.

### Prognostic Assessment With the ICPBS

Univariate Cox analysis was employed to first calculate the prognostic value of every ICP by the R/survival package, and IRGs with *p* < 0.05 were chosen as the seed ICP for Cox LASSO regression. Then, prognostic signatures with the R packages “survminer”, “glment”, and “survival” were identified by multivariate Cox regression. The risk marks for every patient in the training group were calculated according to the following formula: risk mark = expGene1 × βGene1 + expGene2 × βGene2 + expGenen × βGenen (exp, prognostic gene expression level; β, multivariate Cox regression model regression coefficients) (31). All the samples were divided into high- and low-risk mark sets randomly with the mean risk mark as the cutoff value. The survival of every group was analyzed with the Kaplan–Meier method and log-rank test. ROC curves and the corresponding areas under the curve (AUCs) were produced with the R package “survivalROC”.

### Validation of the Prognostic Value of the ICPBS and the Development of a Predictive Model

The ICPBS was validated in various clinical subgroups and histopathological subtypes. Whether the ICPBS is a separate risk factor for CM was evaluated by analyzing the univariate and multivariate Cox regression of the ICPBS and other clinicopathological elements. The indexes of the multivariate Cox regression model (through the R package “rms”, “Hmisc”, “lattice”, “Formula”, and “foreign”) were employed to build the nomogram model. Then, the total risk score was calculated on the basis of every predictor in the nomogram, and the patients were divided into two groups, with the middle risk mark as the cutoff point (32, 33). The C-index, calibration curve, and ROC analysis were adopted to determine the predictive precision and discriminative capacity of the nomogram. The test and validation cohorts adopted a similar analysis process.

### Clinical Use

The clinical effectiveness of the nomogram was determined with decision curve exploration through the quantification of the net benefits at different threshold probabilities in the cohort (34). The net benefit was calculated by subtracting the ratio of all patients with false-positive results from the ratio of patients with true-positive results and by weighing the relative harm of forgoing interventions by comparing with the negative outcomes of an unnecessary intervention (R package “rms” and “rmda”) (35).

### GSEA

The software GSEA v4.0.3 (www.broadinstitute.org/gsea) was used to perform gene set enrichment analysis (GSEA). All of the samples were divided into high- and low-risk score groups on the basis of the middle cutoff. We input the expression profiles of the mRNAs, groups of samples, and background files.

### Differential Analysis

We used the website GEPIA2 (36) to analyze the variation in the expression of ICPBS molecules between CM tissues in TCGA and normal skin tissues in GTEx. We also downloaded two datasets of GSE15605 and GSE46517, which contain the expression matrix of CM tissue and normal skin tissue; then, we calculated the expression difference between the two groups by the Kruskal method and corrected the *p*-value by the Benjamini–Hochberg method (37).

### Statistical Analysis

All the analyses in this study were carried out through R software (version 3.6.3). The immune scores and the abundance of immune cell infiltration of the two groups were compared by the Wilcoxon test. Comparisons among three or more groups were made by one-way ANOVA and Kruskal–Wallis tests. A *p*‐value < 0.05 was regarded statistically significant. All the *p*-values in this study were corrected by the Benjamini–Hochberg method.

## Results

### Unsupervised Cluster Analysis

**Figure 2:**
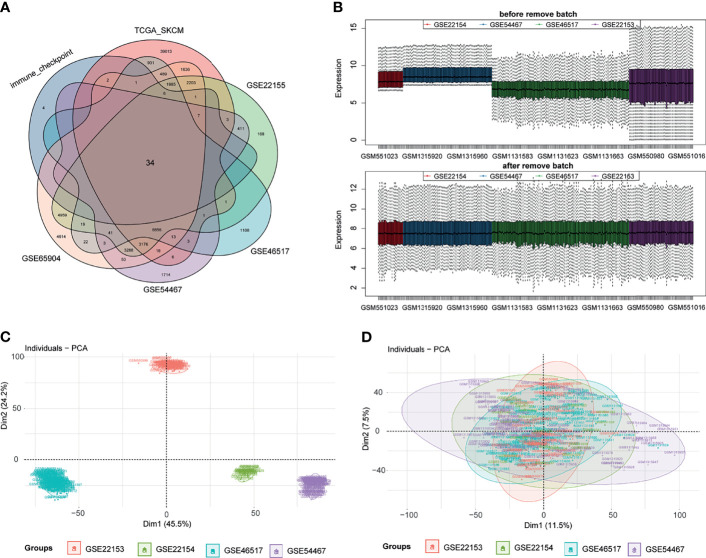
Data processing. Panel **(A)** shows the common immune checkpoint molecules in six datasets in the form of a Venn diagram. Then, we visualize the results of eliminating the batch effect in the form of a box diagram **(B)**. The PCA before and after the merger suggests that we have excellently removed the batch effect **(C, D)**.

**Figure 3:**
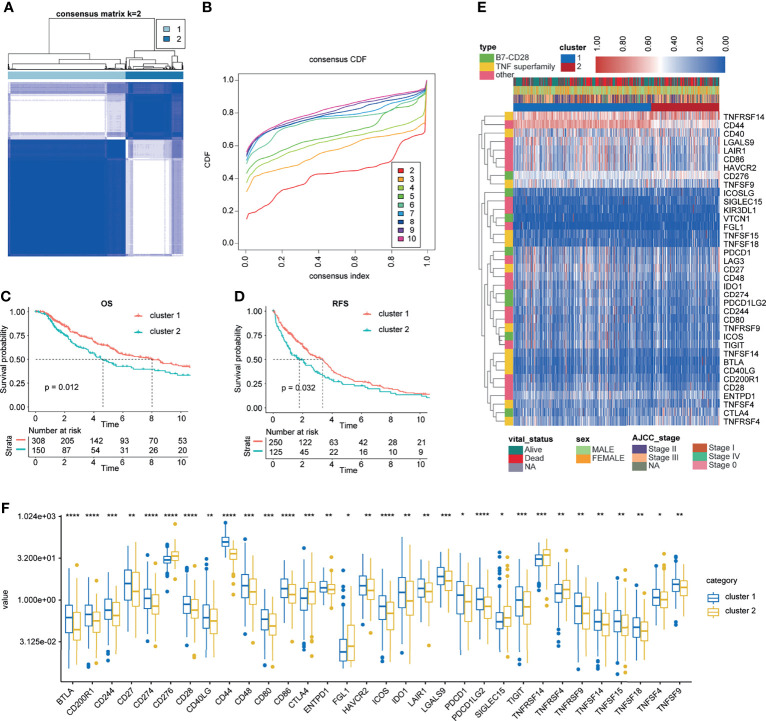
Unsupervised clustering for ICPs. **(A, B)** Classification of TCGA-SKCM into two groups. **(C, D)** Kaplan–Meier curves of OS and RFS in the training cohort based on clustering. **(E)** Landscape of the expression of 34 ICPs in the TCGA-SKCM set. **(F)** The 34 ICPs are significantly differentially expressed between the two clusters. *, **, *** and **** represent P < 0.05, P < 0.01, P < 0.001 and P < 0.0001, respectively.

First, ICPs coincident in the TCGA, GSE22153, GSE22154, GSE46517, GSE54467, and GSE65904 databases were screened (**Figure 2A**). We merged the four datasets (GSE22153, GSE22154, GSE46517, and GSE54467) and removed the batch effect (**Figure 2B**). The PCA before and after the merging suggested that we fully removed the batch effect (**Figures 2C, D**). Based on the ICPs, TCGA was classified into two groups by unsupervised clustering (**Figures 3A, B**). According to the prognostic exploration of the two groups, cluster 1 displayed great benefits in survival and no recurrence (**Figures 3C, D**). A multivariate heat map containing 34 ICP expression levels and clinicopathological data is shown in **Figure 3E**. We found that 34 ICPs were significantly differentially expressed between the two clusters (**Figure 3F**).

### Immune Score and Immune Infiltration Cell Landscape Between the Two Clusters

**Figure 4:**
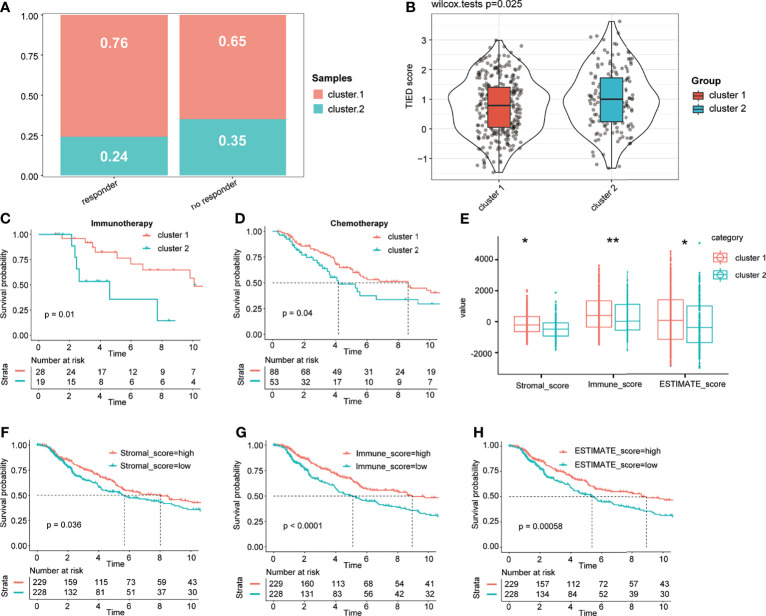
Different responses to treatment between the two groups and immune score landscape in the cohort. Based on the results of TIDE analysis, we compared the efficacy of immune checkpoint block and TIDE score in patients with cluster 1 and cluster 2 **(A, B)**. The KM analysis of the patients who had received immunotherapy and chemotherapy between the two clusters **(C, D)**. Different immune and stromal scores between the two groups **(E)**. Kaplan–Meier analysis of the CM data in the TCGA cohorts showed that OS was significantly shorter in the low-stromal, immune, and ESTIMATE score groups **(F–H)**. * and ** represent P < 0.05, P < 0.01.

Based on the results of TIDE analysis, there were significantly more responses to immune checkpoint inhibition therapy in patients in cluster 1 than in those in cluster 2 (**Figures 4A, B**). Besides, the KM analysis of patients who had received immunotherapy and chemotherapy showed that the response of patients in cluster 1 to immunotherapy and chemotherapy was significantly better than that of patients in cluster 2 (**Figures 4C, D**). Therefore, we named cluster 1 as immunotherapy-sensitive group (ISG) and cluster 2 as immunotherapy-tolerant group (ITG). We further explored the difference in the tumor immune microenvironment between the two groups. In general, the tumor microenvironment includes various cell types (immune, interstitial, and endothelial cells), inflammatory mediators, and extracellular matrix (ECM) molecules (29). For the hidden mechanism between the therapeutic response and tumor immune microenvironment, a set of analytic approaches associated with the immune profile were adopted. Among TCGA cohorts, the stromal, immune, and ESTIMATE scores of the ISG were significantly higher than those of the ITG (**Figure 4E**). According to the Kaplan–Meier exploration of the CM data in TCGA cohorts, OS was notably shorter in the low stromal, immune, and ESTIMATE mark groups (**Figures 4F–H**).

**Figure 5:**
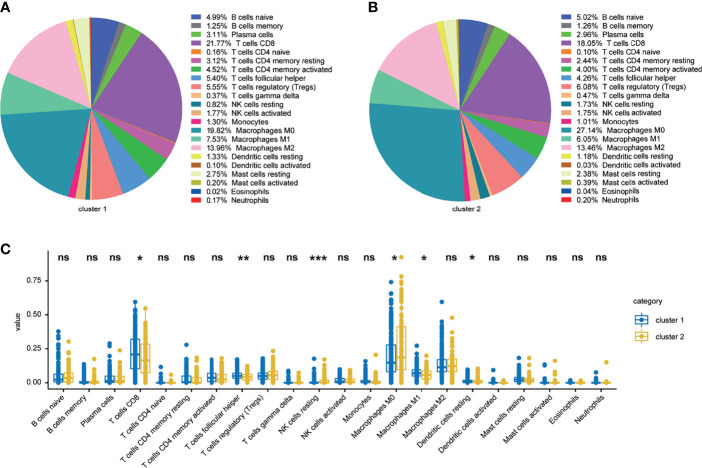
Landscape of 22 infiltrating immune cells in the TCGA cohort. Landscape of infiltrating immune cells in the ISG **(A)** and ITG **(B)** cohort. Specific immune cell infiltration differences between ISG and ITG **(C)**. *, ** and *** represent P < 0.05, P < 0.01, P < 0.001 respectively. Ns means no significance.

Since therapeutic response was closely associated with immune infiltration score, immune cell infiltration variations between ISG and ITG were analyzed. The proportions of 22 and 10 immune cell subtypes were assessed by the CIBERSORT and MCPcounter package on the basis of the expression file, respectively. For further analyses, we used the samples with *p* < 0.05 in the CIBERSORT and MCPcounter exploration outcomes. Most of the differential immune cells between ISG and ITG were found in the CIBERSORT analysis, which were predominantly M0 macrophages, M1 macrophages, follicular helper CD4 T cells, resting NK cells, resting dendritic cells, and CD8 T cells (**Figures 5A–C**). In the MCPcounter analysis, the proportions of CD8 T cells, B lineage, cytotoxic lymphocytes, monocytic lineage, and myeloid dendritic cells in the ISG were higher than those in the ITG (**Figures S1A–C**).

### Identification of Differentially Expressed Genes and the Module Function Annotation

**Figure 6:**
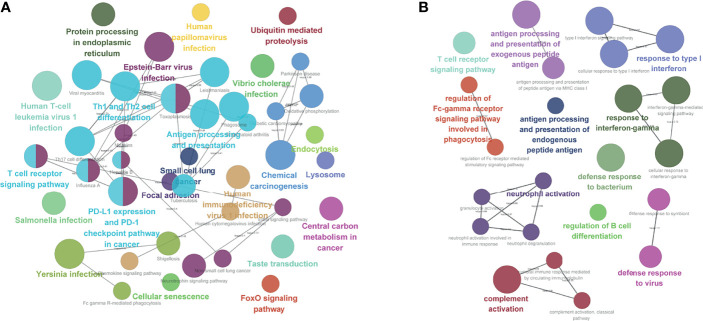
Functional enrichment analysis. **(A)** KEGG pathway analysis. **(B)** Enrichment in immune response-related pathways.

We analyzed the differences in 58,385 genes between ISG and ITG; we found that 1513 genes were upregulated in ISG, while 1,307 genes were upregulated in ITG (**Figures S2A, B**). Functional enrichment analysis was also carried out for the exploration of the GO database terms and KEGG pathways related to the 2,820 significantly differentially expressed genes. According to the outcomes, enrichment in immune response-related pathways included regulation of B-cell differentiation, T-cell receptor signaling pathway, antigen processing, and presentation of endogenous peptide antigen, neutrophil activation involved in immune response, and response to interferon-gamma (**Figure 6B**). Moreover, according to KEGG pathway functional enrichment, PD-L1 expression and PD-1 checkpoint pathway in cancer, T-cell receptor signaling pathway, antigen processing and presentation, Th1 and Th2 cell differentiation, and Th17 cell differentiation were the main pathways related to the genes (**Figure 6A**).

### Construction of the ICPBS for CM

Univariate Cox survival analysis evaluated 34 ICPs in total, and 21 ICPs with *p* < 0.05 were screened and included in the follow-up analyses (**Figure S3A**). According to **Figure S3B**, seven ICPs (lambda value = 7) subject to multivariate Cox regression analysis (**Figure S3C**) were identified by LASSO regression analysis. Ultimately, we identified seven ICPs predicting CM patient prognosis, including CD40, CD80, CD86, ICOSLG, KIR3DL1, LAG3, and TNFSF4. The risk mark of the ICPBS was calculated using the following formula: risk mark = (−0.0017 × expression of CD40) − (0.7615 × expression of CD80) + (0.0092 × expression of CD86) − (1.0053 × expression of ICOSLG) − (0.9862 × expression of KIR3DL1) − (0.0081 × expression of LAG3) − (0.0450 × expression of TNFSF4). The patients in the training and the validation sets were divided into high-risk and low-risk groups, with the middle-risk mark as the cutoff point (**Figures S4A, B**). Moreover, in both the training set and the validation set, the expression of the seven ICPs in the low-risk group was significantly higher than that in the high-risk group (**Figures S4C, D**).

In the TCGA cohort, the patients in the high-risk group had poorer OS and RFS than those in the low-risk group (log‐rank test *p*‐value < 0.0001, **Figures S5A, C**). Similar results were observed in the validation set (**Figure S5D**). Moreover, whether survival predictions made with the ICPBS were accurate in the TCGA and validation cohorts (**Figure S5B, E**) was determined by implementing ROC curve analysis to assess the AUC values for the 3‐year OS for TCGA cohort (AUC = 0.670) and the validation cohort (AUC = 0.784). In order to determine whether the ICPBS is equally effective for the prediction of DSS and DMFS in CM patients, a similar analysis was performed on the test cohort (*n* = 210). According to the Kaplan–Meier curves for DSS and DMFS, patients with high-risk marks had poorer DSS and DMFS than those with low-risk marks (log-rank test *p*-value < 0.001, **Figures S5F, H**). Moreover, according to the outcomes, the AUC for 3-year DSS and DMFS was 0.674 and 0.722, respectively (**Figures S5G, I**).

### Difference in the Expression of Six Molecules in ICPBS Between CM and Normal Skin Tissues

As shown in **Figure S6**, except for KIR3DL1, the other six ICPBS molecules were differentially expressed between CM and normal skin tissues. Specifically, LAG3, CD80, CD86, and TNFSF4 were upregulated (**Figures S6B, C, E, F**), while CD40 and ICOSLG were downregulated in CM tissues compared with normal skin tissues (**Figures S6A, D**). Surprisingly, similar results were observed in GSE15605 (**Figure S7A**) and GSE46517 (**Figure S7B**) datasets.

### Verification of the ICPBS in Various Clinical Subgroups

As the most important element, the pathological stage affects CM patient survival (38), and other factors, including sex and age, are also important (39); hence, CM patients in the training and the verification cohorts were stratified by three clinical features. In all subgroups, including stage I, stage II, stage III, and stage IV; men and women; and older (age ≥ 60 years) and younger (age < 60 years), the patients in the high-risk groups had shorter OS time than those in the low-risk groups (**Figure S8**, *p* < 0.05). Moreover, based on the results of TIDE analysis, we further showed that there were significantly more responses to immune checkpoint inhibitor therapy in patients with low risk than in those with high risk (**Figures S9A, B**). The KM analysis of patients who had received immunotherapy and chemotherapy in both groups showed that the response of the low-risk group to immunotherapy and chemotherapy was better than that of the high-risk group (**Figures S9C, D**).

### Establishment of a Forecast Model by the ICPBS and Clinical Features

**Figure 7:**
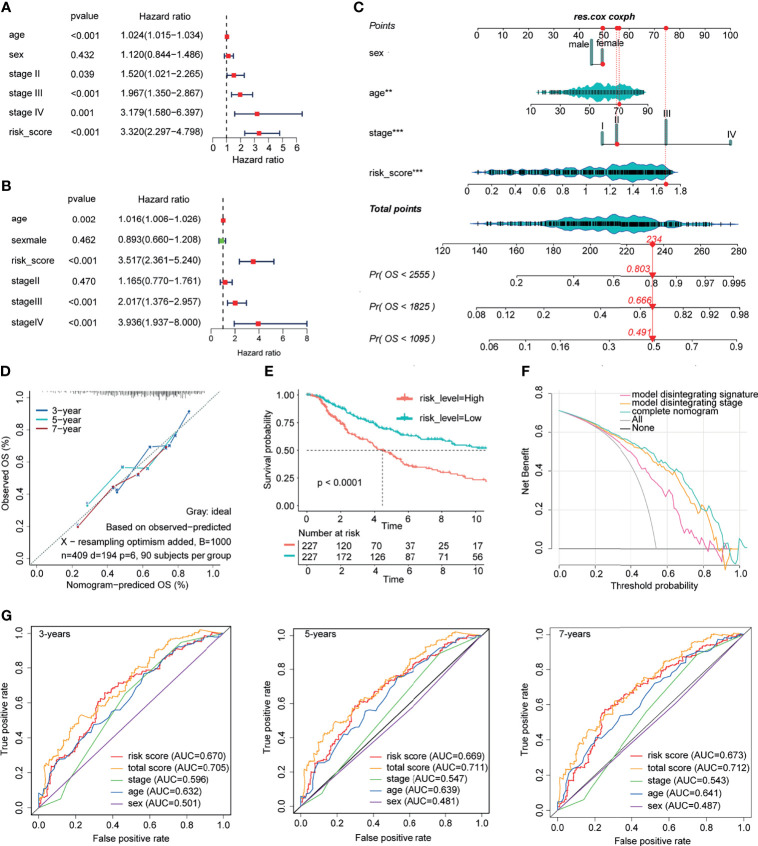
Construction and validation of prognostic nomograph. The univariate and multivariate Cox regression model **(A, B)**. The nomogram model was built by using the coefficients of the multivariate Cox regression model **(C)**. According to the calibration curve, predictive values were consistent with observed values considering the probabilities of 3-year, 5-year, and 7-year OS **(D)**. The AUC values for 3-, 5-, and 7-year survival using the predictive nomogram reached 0.705, 0.711, and 0.702, respectively **(G)**. Kaplan–Meier analysis showed that patients with a high-risk score had an obviously worse OS than patients with a low-risk score **(E)**. Finally, clinical decision analysis (DCA) shows that the clinical benefit rate of the model without stage alone is higher than that of the model without ICBPS alone **(F)**.

The univariate and multivariate Cox regression models included the following clinical candidate predictors: age, sex, stage, and ICPBS (**Figures 7A, B**). ICPBS was an independent risk factor in patients with CM (HR = 3.517, *p* < 0.001). The indexes of the multivariate Cox regression model (**Figure 7C**) were adopted to build the nomogram model. Surprisingly, the C-index of the nomogram for predicting survival was 0.735 (95% CI, 0.733–0.737). According to the calibration curve, the predicted values conformed to the observed values of the probabilities of 3-year, 5-year, and 7-year OS (**Figure 7D**). In the end, the total risk marks were calculated on the basis of every predictor in the nomogram model. The AUC values for 3-, 5-, and 7-year survival using the predictive nomogram were 0.705, 0.711, and 0.702, respectively (**Figure 7G**). Besides, in the ROC analysis, the AUC value of ICPBS was higher than that of stage, and the AUC value of the model was higher than either the ICPBS and AJCC stage. The Kaplan–Meier analysis showed that patients with a high-risk mark had a poorer OS than those with a low-risk mark (log-rank test *p*-value < 0.0001, **Figure 7E**). Finally, clinical decision analysis (DCA) showed that the clinical benefit rate of the model without stage alone was higher than that of the model without ICPBS alone. However, the clinical benefit rate of the complete model with these two factors was higher than that of the model without either of them (**Figure 7F**). These results suggest that ICPBS is superior to the traditional TNM staging system in predicting prognosis. The combined model, including both of these two factors, had the best prognostic ability and predictive stability.

### Verification of the Model in the Verification Cohort

To determine the robustness of this model, similar analyses were conducted in the validation sets. ICPBS was a risk factor in patients with CM (HR = 1.569, *p* = 0.007, **Figures S10A, B**). Next, the indexes of the multivariate Cox regression model were adopted to build the nomogram model. The C-index of the nomogram for predicting survival was 0.757 (95% CI, 0.755–0.759). According to the calibration curve, the predicted values conformed to the observed values of the probabilities of 3‐year, 5‐year, and 7-year OS (**Figure S10C**). In addition, the AUC values shown by the total risk mark for 3-, 5‐, and 7-year survival were 0.810, 0.798, and 0.877, respectively (**Figure S10F**). Moreover, in the ROC analysis, the AUC value of ICPBS was also higher than that of stage, and the AUC value of the combined model was also higher than either of them. According to the Kaplan–Meier curves of OS, patients with high total marks had poorer OS than those with low total marks (log-rank test *p*-value < 0.0001, **Figure S10D**). Ultimately, DCA also showed that the clinical benefit rate of the model without stage alone was higher than that of the model without ICPBS alone and that the clinical benefit rate of the combined model with these two factors was higher than that of the model without either of them (**Figure S10E**).

### GSEA

Twenty KEGG pathways related to the ICPBS, including the MAPK signaling pathway, natural killer cell-mediated cytotoxicity, the P53 signaling pathway, T-cell receptor signaling pathway, B-cell receptor signaling pathway, pathway in cancer, primary immunodeficiency, and intestinal immune network for IgA production (**Figure S11**), were identified by GSEA.

## Discussion

There are several pieces of evidence that immune checkpoint inhibitors are effective in the treatment of CM (40). However, not all patients with CM are sensitive to immunotherapy, and some patients have poor outcomes and serious side effects of immunotherapy, which limits the development of immunotherapy in the treatment of CM (41). A biomarker that could predict the effect of immunotherapy in patients with CM would be greatly appreciated. Therefore, for the first time, here, we analyzed the association between ICPs and immunotherapy and prognosis in patients with CM.

Based on the expression levels of 34 ICPs, we performed unsupervised cluster analysis on TCGA datasets and identified two phenotypes (ITS and ITT). The effect of immunotherapy and prognosis in the ITS group was better than those in the ITT group, indicating that the expression levels of ICPs were closely related to the prognosis and immunotherapy effect in CM patients. Besides, the stromal, immune, and ESTIMATE scores of the ISG were significantly higher than those of the ITG. The proportion of CD8 T cells in the ISG was higher than that in the ITG, both in the CIBERSORT and in the MCPcounter analyses. Jarem et al. found that a higher infiltration abundance of CD8 T cells in CM tissues was associated with better effects of immunotherapy and better prognosis (42). This suggests that the differential expression of immune checkpoints in tumor tissues may affect the effect of immunotherapy and prognosis through CD8 T cells. To further verify our assumption, we performed the functional enrichment analysis based on 2,820 significantly differentially expressed genes between ITS and ITT. The results indicated that the main biological processes involved were neutrophil activation in immune response, regulation of immune effector process, and regulation of immune system process. Moreover, according to KEGG pathway functional enrichment, PD-L1 expression and PD-1 checkpoint pathway in cancer and T-cell receptor signaling pathway were the main pathways related to the genes. These results further indicate that the differentially expressed immune checkpoint molecules affect the prognosis and the effect of immunotherapy mainly by affecting the immunity of the body.

To construct a signature to predict the prognosis and the effect of immunotherapy in patients with CM, we constructed an ICPBS based on seven ICPs (CD40, CD80, CD86, ICOSLG, KIR3DL1, LAG3, and TNFSF4), which could independently predict the prognosis of CM. In addition, ICPBS better predicted the prognosis in patients with CM, compared with the traditional clinicopathological staging system. Moreover, we verified the prognostic value of ICPBS in various molecular and clinical subtypes. It is worth noting that in the ICPBS scoring system, the prognosis and immunotherapy effect of CM patients with high-risk mark were poorer than those of CM patients with low-risk mark, which has great significance for guiding clinical stratified treatment. For patients with low-risk marks, auxiliary chemotherapy and immunotherapy before and after operation can be considered appropriate. In patients with high-risk marks, it is necessary to consider the overall surgical resection and radiotherapy and to make frequent follow-up examinations to monitor the recurrence to allow for timely treatment.

GSEA results showed that a large number of cancer and immune pathways were enriched between the high- and the low-risk groups, suggesting that the variations in survival and immunotherapy between the high- and the low-risk groups may be related to changes in immune status. Finally, we constructed the nomogram based on ICPBS and clinical features such as pathological staging, which can stratify the risk of CM patients and predict the prognosis better than either ICPBS or pathological staging alone.

CD80 (B7-H1) is a member of the B7 co-stimulatory molecule family, which is thought to be involved in regulating cellular and humoral immune responses through activated PD-1 receptors on T and B cells (43). CTLA-4 can downregulate T-cell activation by binding to B7 (CD80/CD86) molecules on antigen-presenting cells (44). Lucas et al. found that the increase in soluble CD80 protein *in vivo* can delay the growth of CM tumor and promote tumor-infiltrating lymphocytes (45). In the present study, CD80 was shown to be an independent protective factor in patients with CM (HR = 0.467, *p* = 0.005). CD80 was highly expressed in both the ITS group and the low-risk score group, suggesting that CD80 does play a significant role in CM and is worth further exploration as a possible therapeutic target.

The results of Natalia et al. showed that the T-cell co-stimulatory ligand (ICOS-L/B7H) can offer a co-stimulatory effect through ICOS to maintain high expression of Foxp3 and CD25 and to stop the expansion of activated Treg. Therefore, the expression of ICOS-L in melanoma cells may promote the activation and amplification of Treg directly in the tumor microenvironment as another mechanism of immune evasion (46). In our study, as an independent indicator of better prognosis in patients with CM (HR = 0.366, *p* = 0.004), the expression of ICOSLG in the low-risk group was higher than that in the high-risk group, suggesting that ICOSLG is the key molecule affecting response to immunotherapy. Further studies on its effect on tumor immune evasion are needed to guide the immunotherapy of CM.

Bakka et al. found that the allogeneic connection of NK cell receptor KIR3DL1 (also known as killer cell immunoglobulin-like receptor, with three Ig domains and a long cytoplasmic tail 1) to HLA-Bw4 inhibited the cytotoxicity of both HLA-B*4403-transfected melanoma and endogenous HLA-Bw4-expressing melanoma. The communication of KIRs with their ligands on melanoma tumor cells may stop tumor cell lysis by NK and gamma delta T-cell clones (47, 48). In this study, KIR3DL1 was shown to be an independent indicator of better prognosis in patients with CM (HR = 0.373, *p* = 0.014). So far, there have been few studies on the relationship between KIR3DL1 and CM. Therefore, the relationship between KIR3DL1 and immunity and prognosis of patients with CM warrants further study.

CD40 is a cell surface member of the tumor necrosis factor receptor superfamily. Once activated, CD40 allows dendritic cells to drive the activation of antitumor T cells and re-educate macrophages to destroy the tumor stroma (49). A number of clinical trials have shown that CD40 agonists are beneficial in the treatment of a variety of tumors, including CM (50). Lymphocyte-activation gene 3 (LAG3; also referred to as CD223) is one of the inhibitory receptors that can facilitate intratumoral T-cell exhaustion (51). Patrice et al. found that MHC class II engagement by its ligand LAG-3 (CD223) contributes to melanoma resistance to apoptosis (52). Although CD40 and LAG3 did not show statistical significance in our multivariate Cox regression analysis, as members of ICPBS, they can also make a good risk stratification and predict the prognosis of patients with CM, which is worth further exploration.

As a co-stimulatory checkpoint protein, TNFSF4 is expressed by some types of immune and non-immune cells and strengthens the antineoplastic activity of T cells (53). Jason et al. found that the low expression of TNFSF4 mRNA was related to the poor prognosis of all patients with melanoma and patients with stage III and IIIc–IV. In the subgroup of patients with low lymphocyte infiltration, the low expression of TNFSF4mRNA was also associated with poor prognosis, suggesting that tumor TNFSF4 was related to prognosis (54). Although TNFSF4 was not an independent protective factor for CM patients in our multivariate Cox regression analysis (HR = 0.956, *p* = 0.082), it was highly expressed in the ITS group and the low-risk score group, suggesting that as a protective factor of CM, it may exert a positive effect on immunotherapy and prognosis of CM.

It has to be mentioned that there are also some limitations in this study. First, the prediction of the therapeutic effect of ICPs in this study lacks verification from clinical samples. Second, this study integrated different datasets from multiple platforms for analysis; although the batch effect was eliminated, there may still be a large bias.

In general, we developed a signature that can stratify the risk and accurately predict the prognosis of patients with CM. Through this ICPBS, clinicians may formulate individual treatment plans, especially while selecting patients who can benefit from immunotherapy, which may increase the survival of CM patients. Based on ICPBS and clinical characteristics, we constructed the nomogram of prognosis in patients with CM, which was superior to ICPBS in efficacy.

## Data Availability Statement

The datasets presented in this study can be found in online repositories. The names of the repository/repositories and accession number(s) can be found in the article/**Supplementary Material**.

## Author Contributions

RM and JL designed the whole study. RM and FY conducted the bioinformatics analyses. RM and TZ wrote the manuscript. JL and TZ helped to improve the manuscript. All authors contributed to the article and approved the submitted version.

## Funding

This work was supported by grants from the Science and Technology Innovation Plan of Hunan province (No. 2018SK2087).

## Conflict of Interest

The authors declare that the research was conducted in the absence of any commercial or financial relationships that could be construed as a potential conflict of interest.

## Publisher’s Note

All claims expressed in this article are solely those of the authors and do not necessarily represent those of their affiliated organizations, or those of the publisher, the editors and the reviewers. Any product that may be evaluated in this article, or claim that may be made by its manufacturer, is not guaranteed or endorsed by the publisher.

## Abbreviations

TCGA, The Cancer Genome Atlas; GEO, Gene Expression Omnibus; ROC, receiver operating characteristic; OS, overall survival; AUC, area under the curve.
