# Supplementary material for: Landscape and Clinical Significance of Immune Checkpoint in Cutaneous Melanoma
Source: Front Immunol. 2021 Dec 24;12:756282. doi: 10.3389/fimmu.2021.756282 (PMC8738081; doi:10.3389/fimmu.2021.756282)
Supplement: Supplementary file 1 [file DataSheet_1.docx]

#cluster

library(ConsensusClusterPlus)

data <- read.table(file = "LOG2.txt", sep = "\t", header = T, stringsAsFactors = F, row.names = 1, check.names = F)

data2 <- data[apply(data, 1, function(x){sum(is.na(x)) < ncol(data)/2}),]

data2 <- as.matrix(data2)

res <- ConsensusClusterPlus(data2, maxK = 10, reps = 1000, pItem = 0.8, pFeature = 1, clusterAlg = "pam", corUse = "complete.obs", seed=123456, plot="pdf", writeTable=T)

write.table(data2, "result.txt")

#PCA

library(psych)

library(reshape2)

library(ggplot2)

library(factoextra)

library(stat)

library(vegan)

exprData <- "before.txt"

sampleFile <- "group.txt"

data <- read.table(exprData, header=T, row.names=NULL,sep="\t")

rownames_data <- make.names(data[,1],unique=T)

data <- data[,-1,drop=F]

rownames(data) <- rownames_data

data <- data[rowSums(data)>0,]

data <- data[apply(data, 1, var)!=0,]

mads <- apply(data, 1, mad)

data <- data[rev(order(mads)),]

dim(data)

data_t <- t(data)

variableL <- ncol(data_t)

if(sampleFile != "") {

sample <- read.table(sampleFile,header = T, row.names=1,sep="\t")

data_t_m <- merge(data_t, sample, by=0)

rownames(data_t_m) <- data_t_m$Row.names

data_t <- data_t_m[,-1]

}

pca <- prcomp(data_t[,1:variableL], scale=T)

print(str(pca))

library(factoextra)

fviz_eig(pca, addlabels = TRUE)

fviz_pca_ind(pca, repel=T)

fviz_pca_ind(pca, col.ind=data_t$conditions, mean.point=F, addEllipses = T, legend.title="Groups")

fviz_pca_ind(pca, col.ind=data_t$conditions, mean.point=F, addEllipses = T, legend.title="Groups", ellipse.type="confidence", ellipse.level=0.95)

fviz_pca_var(pca, select.var = list(cos2 = 0.99), repel=T, col.var = "cos2", geom.var = c("arrow", "text") )

fviz_pca_var(pca, select.var= list(cos2 = 10), repel=T, col.var = "contrib")

#Differential analysis

library(limma)

library(edgeR)

counts <- read.table(file = "conut_all.txt", sep = "\t", header = TRUE, row.names = 1, stringsAsFactors = FALSE)

dge <- DGEList(counts = counts)

dge <- calcNormFactors(dge)

logCPM <- cpm(dge, log=TRUE, prior.count=3)

group_list <- factor(c(rep("control",2), rep("siSUZ12",2)))

design <- model.matrix(~group_list)

colnames(design) <- levels(group_list)

rownames(design) <- colnames(counts)

fit <- lmFit(logCPM, design)

fit <- eBayes(fit, trend=TRUE)

output <- topTable(fit, coef=2,n=Inf)

sum(output$adj.P.Val<0.05)

#GSEA

library(clusterProfiler)

library(enrichplot)

library(ReactomePA)

library(data.table)

library("org.Hs.eg.db")

genelist_input <- fread(file="data.txt", header = T, sep='\t', data.table = F)

inputfile="gsea.txt"

gene_symbol=read.table(inputfile,sep="\t",check.names=F,header=T)

gene_name=as.vector(gene_symbol[,1])

foldChange=as.character(gene_symbol[,2])

geneID <- mget(gene_name, org.Hs.egSYMBOL2EG, ifnotfound=NA)

geneID <- as.character(geneID)

data=cbind(gene_symbol,entrezID=geneID)

head(genelist_input)

write.csv(data,"data.csv",row.names =F)

geneList = genelist_input[,2]names(geneList) = as.character(genelist_input[,1])geneList = sort(geneList, decreasing = TRUE)

Go_Reactomeresult <- gsePathway(geneList, nPerm = 1000, minGSSize = 10, maxGSSize = 1000, pvalueCutoff=0.05)

gseaplot2(Go_Reactomeresult, 1:3, pvalue_table = TRUE)

#unicox

library(survival)

inputfile="immueScore_XCELL.txt"

lncRNA<-read.table(inputfile,header=T,sep="\t",row.names = 1,check.names = F)

lncRNAEXP=lncRNA[,3:ncol(lncRNA)]

lncRNA=cbind(lncRNA[,1:2],lncRNAEXP)

coxR=data.frame()

coxf<-function(x){

fmla1 <- as.formula(Surv(OS,vital_status)~lncRNA[,x])

mycox <- coxph(fmla1,data=lncRNA)

}

for(a in colnames(lncRNA[,3:ncol(lncRNA)])){

mycox=coxf(a)

coxResult = summary(mycox)

coxR=rbind(coxR,cbind(lncRNAname=a,

HR=coxResult$coefficients[,"exp(coef)"],

HR.95L=coxResult$conf.int[,"lower .95"],

HR.95H=coxResult$conf.int[,"upper .95"],

P=coxResult$coefficients[,"Pr(>|z|)"]))}

write.table(coxR,"XCELL.txt",sep="\t",row.names=F,quote=F)

#LASSO

library(glmnet)

library(survival)

inputfile="lasso.txt"

lncRNA<-read.table(inputfile,header=T,sep="\t",row.names = 1,check.names = F,stringsAsFactors = F)

lncRNAEXP=lncRNA[,3:ncol(lncRNA)]

lncRNA=cbind(lncRNA[,1:2],lncRNAEXP)

lncRNA[,"OS"]=lncRNA[,"OS"]/365

v1<-as.matrix(lncRNA[,c(3:ncol(lncRNA))])

v2 <- as.matrix(Surv(lncRNA$OS,lncRNA$vital_status))

myfit <- glmnet(v1, v2, family = "cox")

pdf("lambda.pdf")

plot(myfit, xvar = "lambda", label = TRUE)

dev.off()

myfit2 <- cv.glmnet(v1, v2, family="cox")

pdf("min.pdf")

plot(myfit2)

abline(v=log(c(myfit2$lambda.min,myfit2$lambda.1se)),lty="dashed")

dev.off()

myfit2$lambda.1se

coe <- coef(myfit, s = myfit2$lambda.1se)

act_index <- which(coe != 0)

act_coe <- coe[act_index]

row.names(coe)[act_index]

#mulcox

library(survival)

inputfile="mulcox.txt"

lncRNA<-read.table(inputfile,header=T,sep="\t",row.names = 1,check.names = F,stringsAsFactors = F)

lncRNAEXP=lncRNA[,3:ncol(lncRNA)]

lncRNA=cbind(lncRNA[,1:2],lncRNAEXP)

lncRNA[,"OS"]=lncRNA[,"OS"]/365

fmla1 <- as.formula(Surv(OS,vital_status)~.)

mycox <- coxph(fmla1,data=lncRNA)

risk_score<-predict(mycox,type="risk",newdata=lncRNA)

risk_level<-as.factor(ifelse(risk_score>median(risk_score),"High","Low"))

write.table(cbind(id=rownames(cbind(lncRNA[,1:2],risk_score,risk_level)),cbind(lncRNA[,1:2],risk_score,risk_level)),"risk_score.txt",sep="\t",quote=F,row.names=F)

summary(mycox)

#nomogram

library(regplot)

library("survival")

library("survminer")

tcga<-read.table("clinical.txt",header=T,sep="\t")

tcga$risk_level <- as.factor(ifelse(tcga$risk_level==1,"low","hig"))

res.cox <- coxph(Surv(OS, vital_status) ~ age + sex + stage + risk_score, data = tcga)

res.cox

summary(res.cox)

nom1<-regplot(res.cox, clickable=TRUE,

points=TRUE, rank="sd",failtime = c(1095,1825,2555),prfail = T)

nom2<-regplot(res.cox,observation=tcga[5,], clickable=TRUE,

points=TRUE, rank="sd",failtime = c(1095,1825,2555),droplines=T,prfail = T,

other=(list(bvcol="red",sq="green",obscol="blue")))

#calibriation

library(survival)

library(rms)

library(foreign)

tcga<-read.table("clinical.txt",header=T,sep="\t")

ddist <- datadist(tcga)

options(datadist='ddist')

cox1 <- cph(Surv(OS,vital_status) ~ age + sex + stage + risk_score,surv=T,x=T, y=T,time.inc = 1*365*3,data=tcga)

cal3 <- calibrate(cox1, cmethod="KM", method="boot", u=1*365*3, m=49 , B=1000)

cox2 <- cph(Surv(OS,vital_status) ~ age + sex + stage + risk_score,surv=T,x=T, y=T,time.inc = 1*365*5,data=tcga)

cal5 <- calibrate(cox2, cmethod="KM", method="boot", u=1*365*5, m=49 , B=1000)

cox3 <- cph(Surv(OS,vital_status) ~ age + sex + stage + risk_score,surv=T,x=T, y=T,time.inc = 1*365*7,data=tcga)

cal7 <- calibrate(cox2, cmethod="KM", method="boot", u=1*365*7, m=49 , B=1000)

pdf("calibration_compare.pdf",width = 8,height = 8)

plot(cal3,lwd = 2,lty = 0,errbar.col = c("#2166AC"),

bty = "l", #只画左边和下边框

xlim = c(0,1),ylim= c(0,1),

xlab = "Nomogram-prediced OS (%)",ylab = "Observed OS (%)",

col = c("#2166AC"),

cex.lab=1.2,cex.axis=1, cex.main=1.2, cex.sub=0.6)

lines(cal3[,c('mean.predicted',"KM")],

type = 'b', lwd = 1, col = c("#2166AC"), pch = 16)

mtext("")

plot(cal5,lwd = 2,lty = 0,errbar.col = c("#00CED1"),

xlim = c(0,1),ylim= c(0,1),col = c("#00CED1"),add = T)

lines(cal5[,c('mean.predicted',"KM")],

type = 'b', lwd = 1, col = c("#00CED1"), pch = 16)

plot(cal7,lwd = 2,lty = 0,errbar.col = c("#B2182B"),

xlim = c(0,1),ylim= c(0,1),col = c("#B2182B"),add = T)

lines(cal7[,c('mean.predicted',"KM")],

type = 'b', lwd = 1, col = c("#B2182B"), pch = 16)

abline(0,1, lwd = 2, lty = 3, col = c("#224444"))

legend("topleft",

legend = c("3-year","5-year","7-year"),

col =c("#2166AC","#00CED1","#B2182B"),

lwd = 2,

cex = 1.2,

bty = "n")

dev.off()

#risk score

library(survival)

library(rms)

library(foreign)

tcga<-read.table("clinical.txt",header=T,sep="\t")

ddist <- datadist(tcga)

options(datadist='ddist')

cox2 <- coxph(Surv(OS,vital_status) ~ age + sex + stage + risk_score,data=tcga)

risk_score<-predict(cox2,type="risk",newdata=tcga)

risk_level<-as.vector(ifelse(risk_score>median(risk_score),"High","Low"))

write.table(cbind(id=rownames(cbind(tcga[,1:2],risk_score,risk_level)),cbind(tcga[,1:2],risk_score,risk_level)),"risk_score.txt",sep="\t",quote=F,row.names=F)

#ROC

library(survival)

library(timeROC)

lncRNA<-read.table("risk_score.txt",header=T,sep="\t")

predict_1_year<- 1

predict_2_year<- 2

predict_3_year<- 3

predict_4_year<- 4

predict_5_year<- 5

ROC<-timeROC(T=lncRNA$OS,delta=lncRNA$vital_status,

marker=lncRNA$risk_score,cause=1,

weighting="marginal",

times=c(predict_1_year,predict_2_year,predict_3_year,predict_4_year,predict_5_year),ROC=TRUE)

pdf("ROC11.pdf")

plot(ROC,time=predict_1_year,title=FALSE,lwd=3)

plot(ROC,time=predict_2_year,col="turquoise",add=TRUE,title=FALSE,lwd=3)

plot(ROC,time=predict_3_year,col="purple",add=TRUE,title=FALSE,lwd=3)

plot(ROC,time=predict_4_year,col="magenta",add=TRUE,title=FALSE,lwd=3)

plot(ROC,time=predict_5_year,col="green",add=TRUE,title=FALSE,lwd=3)

legend("bottomright",

c(paste("AUC of 1 year survival: ",round(ROC$AUC[1],3)),

paste("AUC of 2 year survival: ",round(ROC$AUC[2],3)),

paste("AUC of 3 year survival: ",round(ROC$AUC[3],3)),

paste("AUC of 4 year survival: ",round(ROC$AUC[4],3)),

paste("AUC of 5 year survival: ",round(ROC$AUC[5],3))),col=c("red","turquoise","purple","magenta","green"),lwd=2)

dev.off()

#KM

library(survival)

library(survminer)

inputdata<- read.table("risk_score.txt",header=T,sep="\t")

fit <- survfit(Surv(OS,vital_status)~ TMB,data = inputdata)

ggsurvplot(

fit,

risk.table = TRUE,

pval = TRUE,

conf.int = FALSE,

surv.median.line="hv",

xlim = c(0,20),

break.time.by = 5,

risk.table.y.text.col = T,

risk.table.y.text = FALSE

)

#DCA

library(survival)

library(timeROC)

setwd("E:\\谷歌下载\\芯片\\生信最新文章\\黑色素瘤\\衰老\\validation\\nom")

lncRNA<-read.table("risk_score.txt",header=T,sep="\t")

predict_1_year<- 1

predict_2_year<- 2

predict_3_year<- 3

predict_4_year<- 4

predict_5_year<- 5

ROC<-timeROC(T=lncRNA$OS,delta=lncRNA$vital_status,

marker=lncRNA$risk_score,cause=1,

weighting="marginal",

times=c(predict_1_year,predict_2_year,predict_3_year,predict_4_year,predict_5_year),ROC=TRUE)

pdf("ROC11.pdf")

plot(ROC,time=predict_1_year,title=FALSE,lwd=3)

plot(ROC,time=predict_2_year,col="turquoise",add=TRUE,title=FALSE,lwd=3)

plot(ROC,time=predict_3_year,col="purple",add=TRUE,title=FALSE,lwd=3)

plot(ROC,time=predict_4_year,col="magenta",add=TRUE,title=FALSE,lwd=3)

plot(ROC,time=predict_5_year,col="green",add=TRUE,title=FALSE,lwd=3)

legend("bottomright",

c(paste("AUC of 1 year survival: ",round(ROC$AUC[1],3)),

paste("AUC of 2 year survival: ",round(ROC$AUC[2],3)),

paste("AUC of 3 year survival: ",round(ROC$AUC[3],3)),

paste("AUC of 4 year survival: ",round(ROC$AUC[4],3)),

paste("AUC of 5 year survival: ",round(ROC$AUC[5],3))),col=c("red","turquoise","purple","magenta","green"),lwd=2)

dev.off()

#maftool

library(maftools)

laml.maf = read.csv("TCGA.csv",header=TRUE)

laml = read.maf(maf = laml.maf)

laml

write.mafSummary(maf = laml, basename = 'laml')

plotmafSummary(maf = laml, rmOutlier = TRUE, addStat = 'median', dashboard = TRUE, titvRaw = FALSE)

oncoplot(maf = laml, top=30)

oncostrip(maf = laml, genes = c())

oncostrip(maf = laml, genes = c())

,oncostrip(maf = laml, genes = c())

laml.titv = titv(maf = laml, plot = FALSE, useSyn = TRUE)

#plot titv summary

plotTiTv(res = laml.titv)

rainfallPlot(maf = laml, detectChangePoints = TRUE, pointSize = 0.6)

laml.mutload = tcgaCompare(maf = laml, cohortName = 'CLON-2')

geneCloud(input = laml, minMut = 15)

Interact <- somaticInteractions(maf = laml, top = 25, pvalue = c(0.05, 0.1))

Interact$gene_sets

Our_maf <- read.csv("Our_maf.csv",header=TRUE)

our_maf = read.maf(maf = Our_maf)

pt.vs.rt <- mafCompare(m1 = laml, m2 = our_maf, m1Name = 'LIHC', m2Name = 'OUR', minMut = 5)

print(pt.vs.rt)

forestPlot(mafCompareRes = pt.vs.rt, pVal = 0.01, color = c('royalblue', 'maroon'), geneFontSize = 0.8)

OncogenicPathways(maf = laml)

PlotOncogenicPathways(maf = laml, pathways = "PI3K")

#risk heatmap

library(pheatmap)

bioRiskPlot=function(inputFile=null,riskScoreFile=null,survStatFile=null,heatmapFile=null){

rt=read.table(inputFile,sep="\t",header=T,row.names=1,check.names=F)

rt=rt[order(rt$riskScore),]

riskClass=rt[,"risk"]

lowLength=length(riskClass[riskClass=="Low"])

highLength=length(riskClass[riskClass=="High"])

line=rt[,"riskScore"]

line[line>10]=10

pdf(file=riskScoreFile,width = 10,height = 3.5)

plot(line, type="p", pch=20,

xlab="Patients (increasing risk socre)", ylab="Risk score",

col=c(rep("blue",lowLength),rep("red",highLength)) )

abline(h=median(rt$riskScore),v=lowLength,lty=2)

legend("topleft", c("High risk", "low Risk"),bty="n",pch=19,col=c("red","blue"),cex=1.2)

dev.off()

color=as.vector(rt$fustat)

color[color==1]="red"

color[color==0]="blue"

pdf(file=survStatFile,width = 10,height = 3.5)

plot(rt$futime, pch=19,

xlab="Patients (increasing risk socre)", ylab="Survival time (years)",

col=color)

legend("topleft", c("dead", "alive"),bty="n",pch=19,col=c("red","blue"),cex=1.2)

abline(v=lowLength,lty=2)

dev.off()

rt1=rt[c(3:(ncol(rt)-2))]

rt1=log2(rt1+1)

rt1=t(rt1)

annotation=data.frame(type=rt[,ncol(rt)])

rownames(annotation)=rownames(rt)

pdf(file=heatmapFile,width = 10,height = 3.5)

pheatmap(rt1, annotation=annotation, cluster_cols = FALSE,fontsize_row=11, show_colnames = F,fontsize_col=3,color = colorRampPalette(c("blue", "white", "red"))(50) )

dev.off()

}

bioRiskPlot(inputFile="TCGA.txt",riskScoreFile="TCGA.riskScore.pdf",survStatFile="TCGA.survStat.pdf",heatmapFile = "TCGAheatmap.pdf")

bioRiskPlot(inputFile="GEO.txt",riskScoreFile="GEO.riskScore.pdf",survStatFile="GEO.survStat.pdf",heatmapFile = "GEOheatmap.pdf")

#Forest plot

setwd("")

rt <- read.table("mulcox.txt",header=T,sep="\t",row.names=1,check.names=F)

gene <- rownames(rt)

hr <- sprintf("%.3f",rt$"HR")

hrLow <- sprintf("%.3f",rt$"HR.95L")

hrHigh <- sprintf("%.3f",rt$"HR.95H")

Hazard.ratio <- paste0(hr,"(",hrLow,"-",hrHigh,")")

pVal <- ifelse(rt$pvalue<0.001, "<0.001", sprintf("%.3f", rt$pvalue))

pdf(file="forest-mulcox.pdf", width =6,height =5)

n <- nrow(rt)

nRow <- n+1

ylim <- c(1,nRow)

layout(matrix(c(1,2),nc=2),width=c(3,2))

xlim = c(0,3)

par(mar=c(4,2.5,2,1))

plot(1,xlim=xlim,ylim=ylim,type="n",axes=F,xlab="",ylab="")

text.cex=0.8

text(0,n:1,gene,adj=0,cex=text.cex)

text(1.5-0.5*0.2,n:1,pVal,adj=1,cex=text.cex);text(1.5-0.5*0.2,n+1,'pvalue',cex=text.cex,font=2,adj=1)

text(3,n:1,Hazard.ratio,adj=1,cex=text.cex);text(3,n+1,'Hazard ratio',cex=text.cex,font=2,adj=1,)

par(mar=c(4,1,2,1),mgp=c(2,0.5,0))

xlim = c(0,max(as.numeric(hrLow),as.numeric(hrHigh)))

plot(1,xlim=xlim,ylim=ylim,type="n",axes=F,ylab="",xaxs="i",xlab="Hazard ratio")

arrows(as.numeric(hrLow),n:1,as.numeric(hrHigh),n:1,angle=90,code=3,length=0.05,col="darkblue",lwd=2.5)

abline(v=1,col="black",lty=2,lwd=2)

boxcolor = ifelse(as.numeric(hr) > 1, 'red', 'green')

points(as.numeric(hr), n:1, pch = 15, col = boxcolor, cex=1.3)

axis(1)

dev.off()
