# Supplementary material for: Landscape and Clinical Significance of Immune Checkpoint in Cutaneous Melanoma
Source: Front Immunol. 2021 Dec 24;12:756282. doi: 10.3389/fimmu.2021.756282 (PMC8738081; doi:10.3389/fimmu.2021.756282)
Supplement: Supplementary file 3 [file DataSheet_3.docx]

**Supplementary tables**

| **Table-S1: Basic information of datasets included in this study.** | | | | |
| --- | --- | --- | --- | --- |
| **Accession number /Source** | **Platform** | **Number of  patients** | **Stage** | **Survival data** |
| GEO: GSE22153 | GPL6102 | 57 | III:3 IV:54 | OS |
| GEO: GSE22154 | GPL6947 | 22 | IV:22 | OS |
| GEO: GSE46517 | GPL96 | 121 | I/II:27 III/IV:55 | OS |
| GEO: GSE54467 | GPL6884 | 79 | I/II:58 III/IV:24 | OS |
| GEO: GSE65904 | GPL10558 | 214 | NA | DFS/DMFS |
| GEO: GSE15605 | GPL570 | 74 | NA | NA |
| TCGA: SKCM | Illumina RNAseq | 469 | I/II:147 III/IV:240 | OS/DFS |


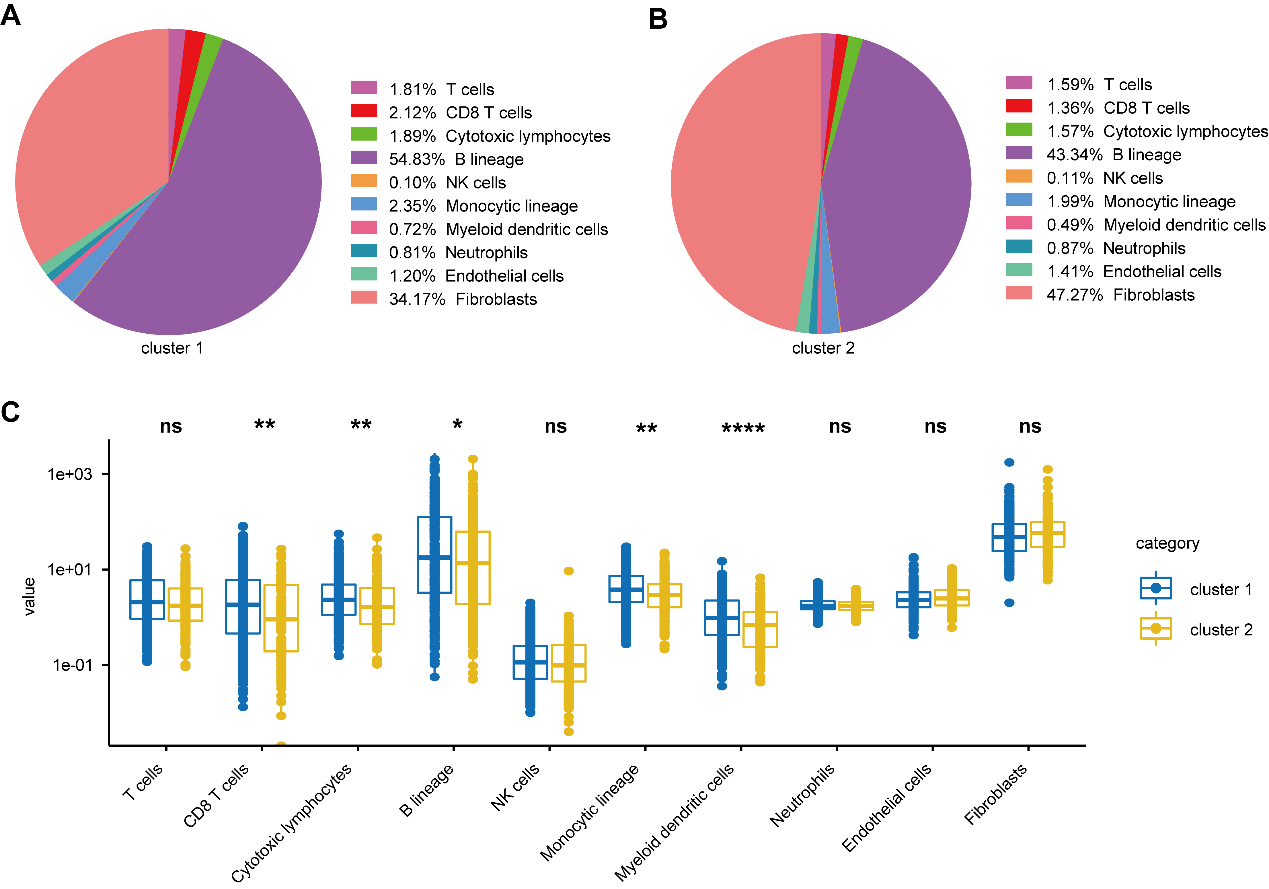


**Figure S1**. **Landscape of 22 infiltrating immune cells in the TCGA cohort.** Landscape of infiltrating immune cells in ISG (**A**) and ITG (**B**) cohort. specific immune cell infiltration differences between ISG and ITG (**C**).


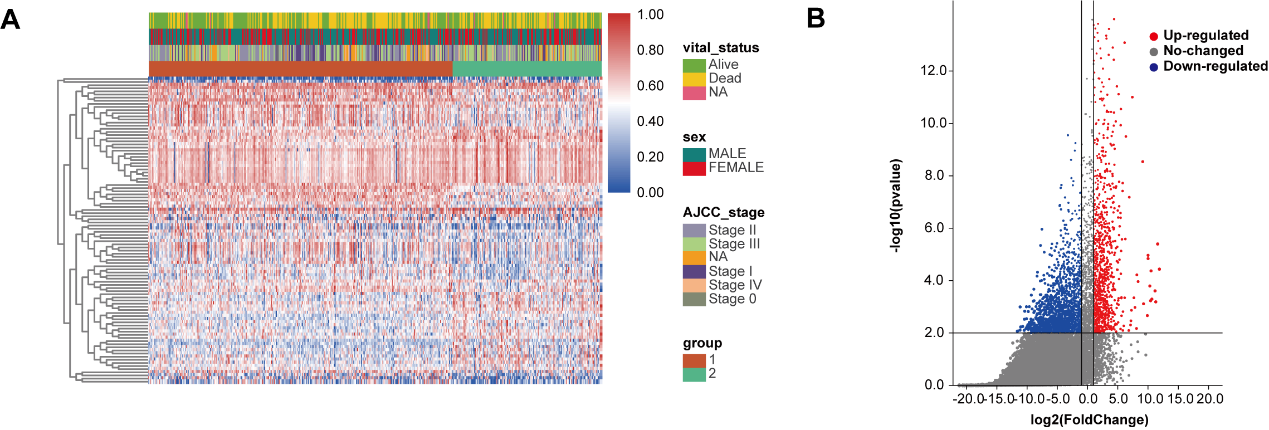


**Figure S2**. **Identification of differentially expressed genes. panel A** and **B** shows the differential gene heatmap and volcanol between ISG and ITG.

**
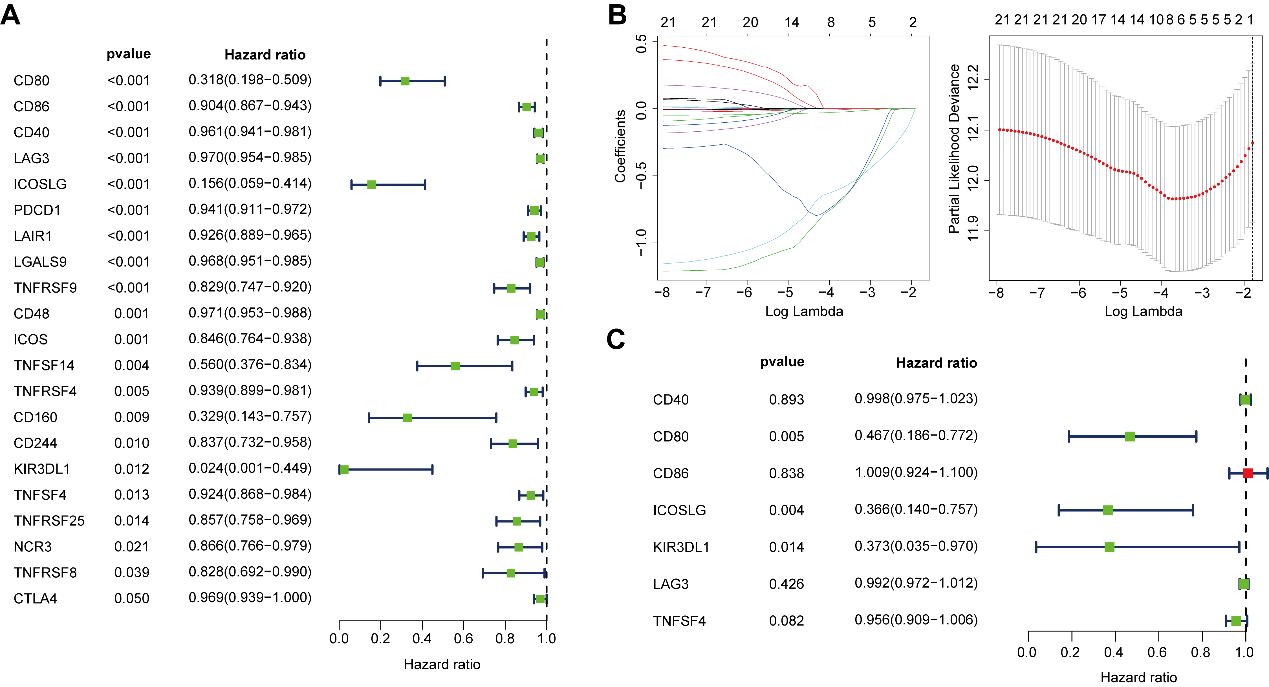
**

**Figure S3**. **Construction of the ICBPS for CM**. A total of 34 ICPs were evaluated by univariate Cox survival analysis, and 21 ICPs with P < 0.05 were filtered out and included in subsequent analyses (**A**). As shown in **panel B**, LASSO regression analysis identified 7 ICPs (lambda value=7) that were subjected to multivariate Cox regression analysis (**C**).

**
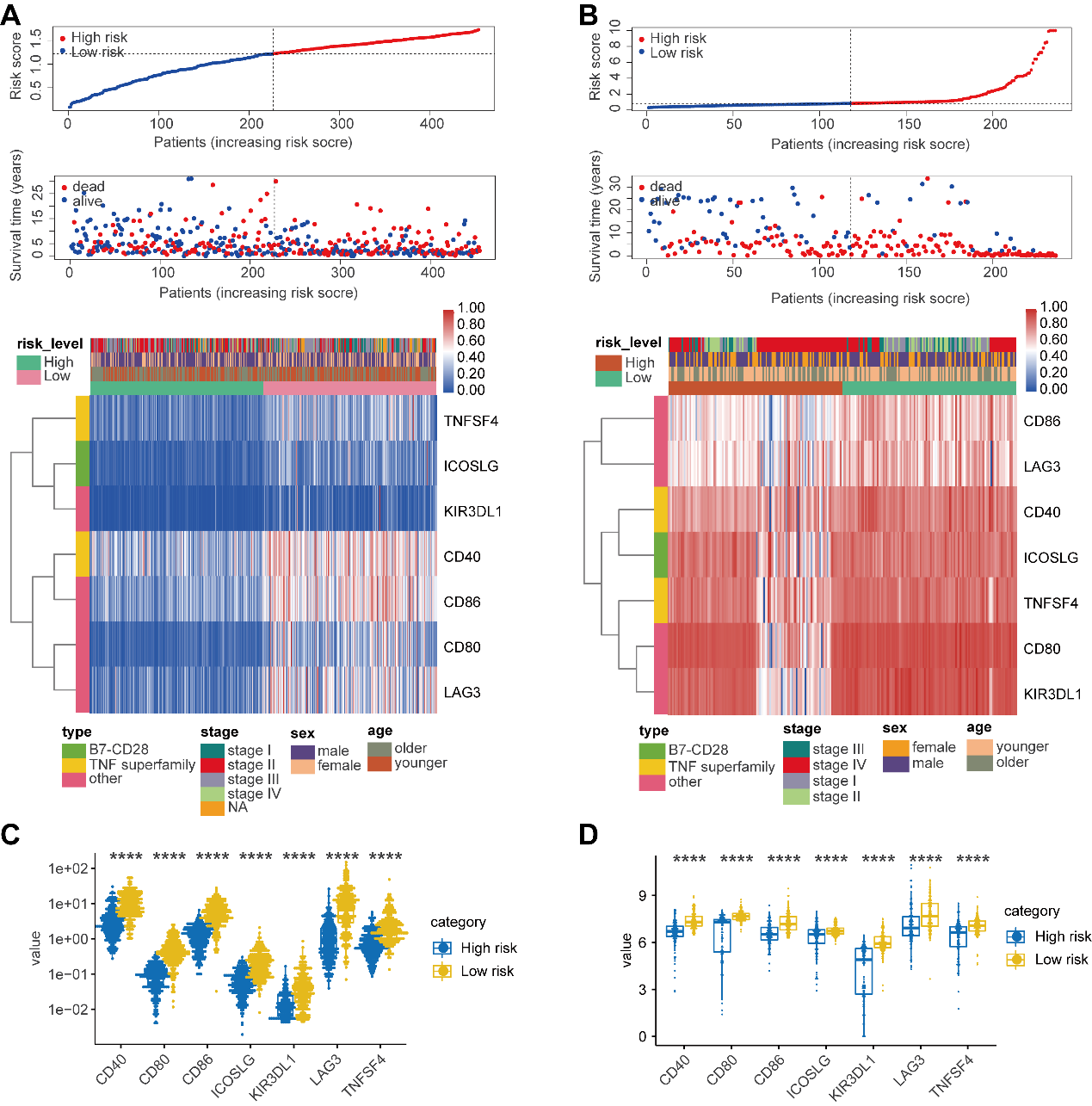
**

**Figure S4**. **The distribution of risk score, recurrence status, and gene expression panel.** Correlation between ICBPS and the OS of patients in the training (**A**) and test (**B**) cohort. The distribution of risk scores (upper), recurrence time (middle), and ICPS expression levels (lower). The black dotted lines represent the median risk score cut-off dividing patients into low- and high-risk score groups. The red dots and lines represent the patients in the high-score groups. The green dots and lines represent the patients in the low-score groups. What's more, in the training set and validation set, the expression of 7 ICP in the low- risk group was significantly higher than that in the high-risk group (**C and D**).

**
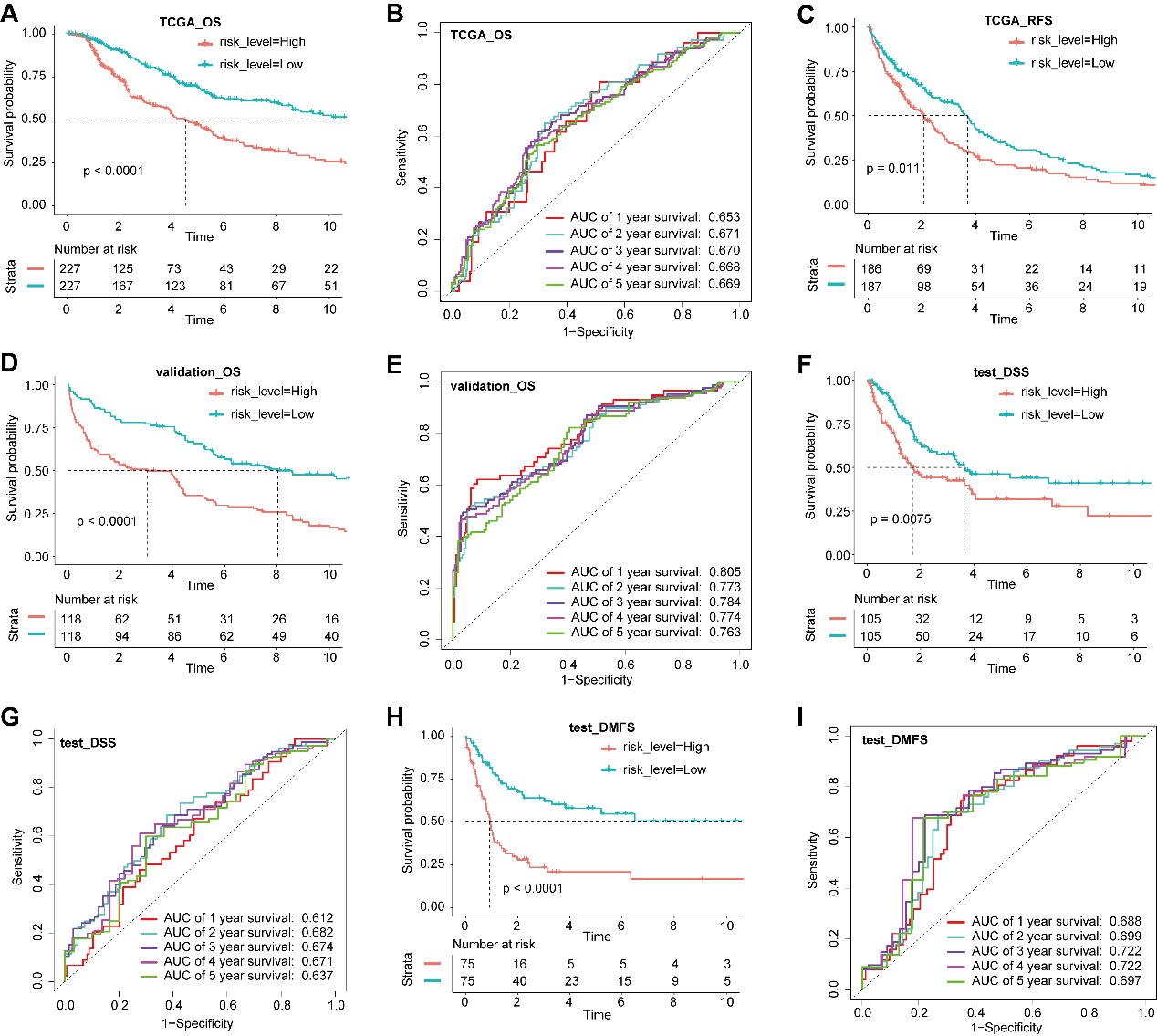
**

**Figure S5**. **Validation of the ICBPS for CM**. In the TCGA cohort, the patients in the high-risk group had significantly worse OS and RFS than those in the low-risk group (**A and C**). Similar results can be observed in the validation set (**D**). In addition, ROC curve analysis was implemented to determine whether survival predictions made with the ICBPS were accurate in the TCGA and validation cohorts (**B and E**). The Kaplan-Meier curves for DSS and DMFS suggested that the patients with high-risk scores had significantly worse DSS and DMFS than those with low-risk scores (**F and H**). In addition, the results showed that the AUC for 3-year DSS and DMFS reached 0.674 and 0.722, respectively (**G and I**).


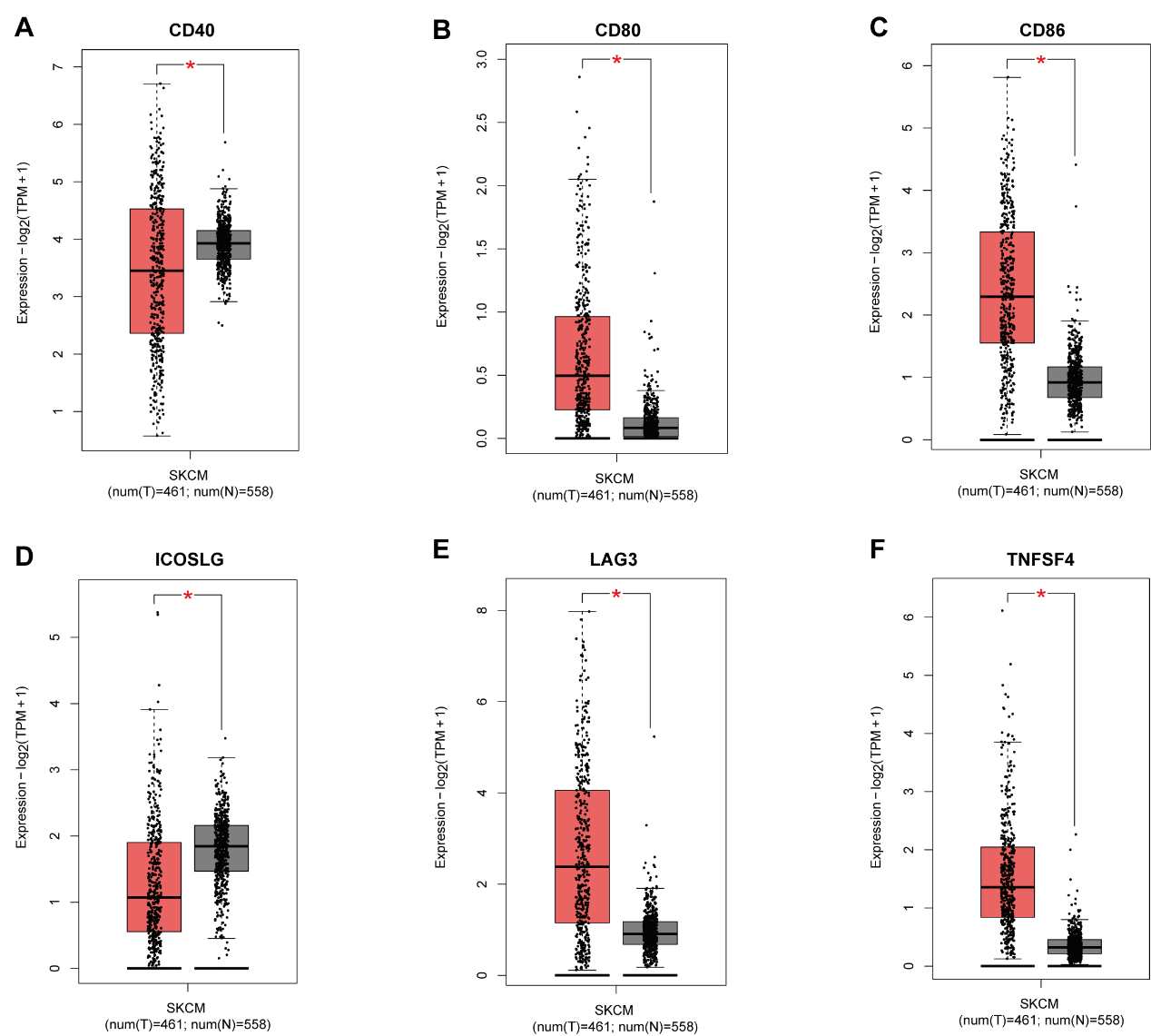


**Figure S6**. the difference in the expression of ICBPS molecules between CM tissues in TCGA and normal skin tissues in GTEx. Red represents tumor tissue, gray represents normal tissue.


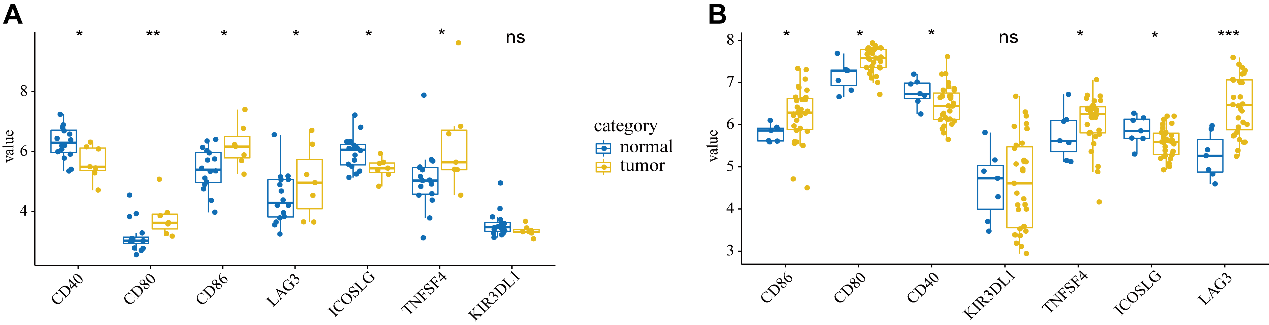


**Figure S7**. the difference in the expression of ICBPS molecules between CM tissues and normal skin tissues in GSE15605 (**A**) and GSE46517 (**B**). Yellow represents tumor tissue, blue represents normal tissue.

**
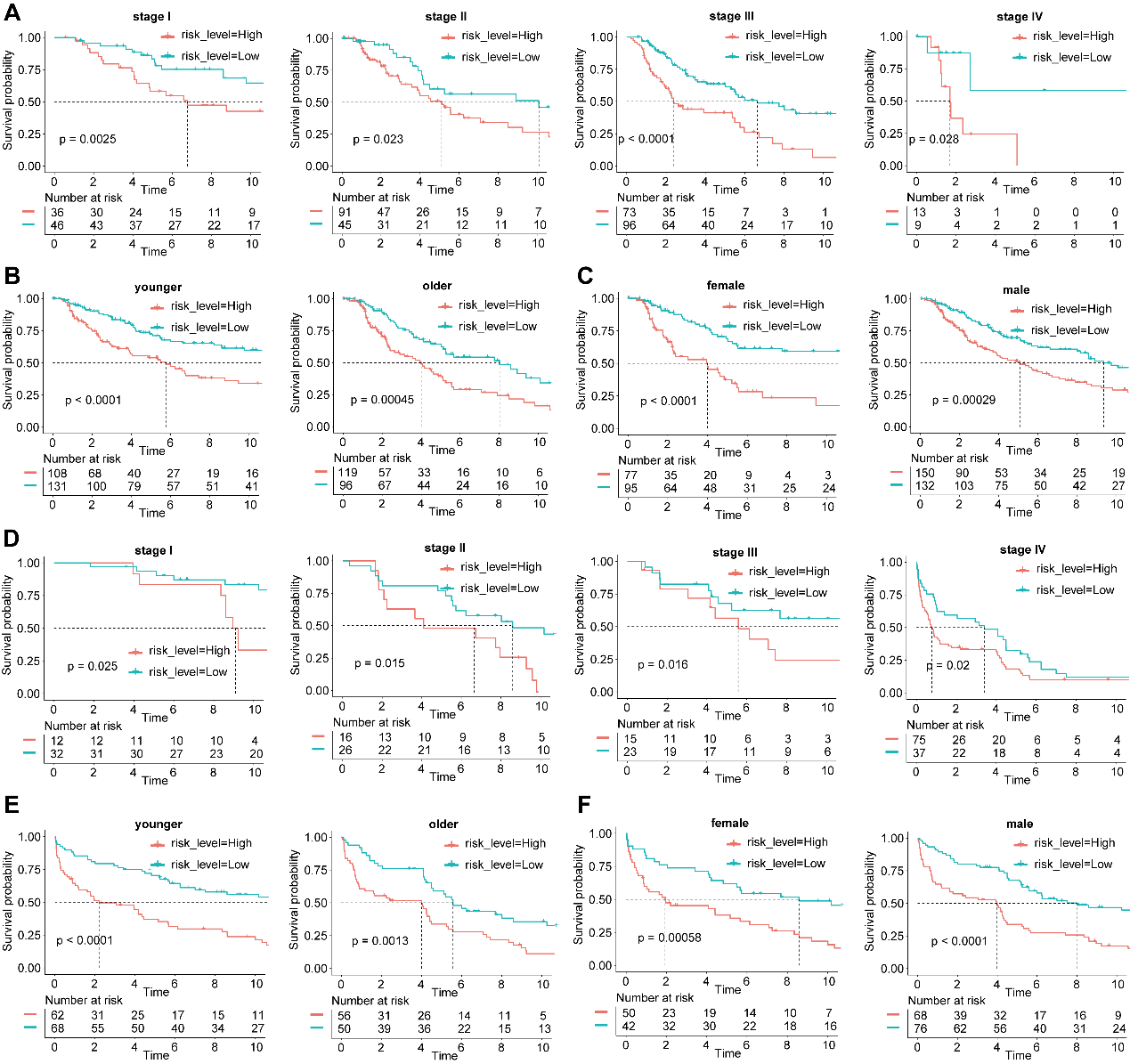
**

**Figure S8**. **Validation of the ICBPS in different clinical subgroups.** in all subgroups of TCGA-SKCM cohort including stage I, stage II, stage III, and stage IV (**A**), males and females (**C**), and older (age ≥ 60 years) and younger (age < 60 years) (**B**), the patients in the high-risk groups had shorter OS times than those in the low-risk groups. There are similar results in the validation set (D, E, and F).


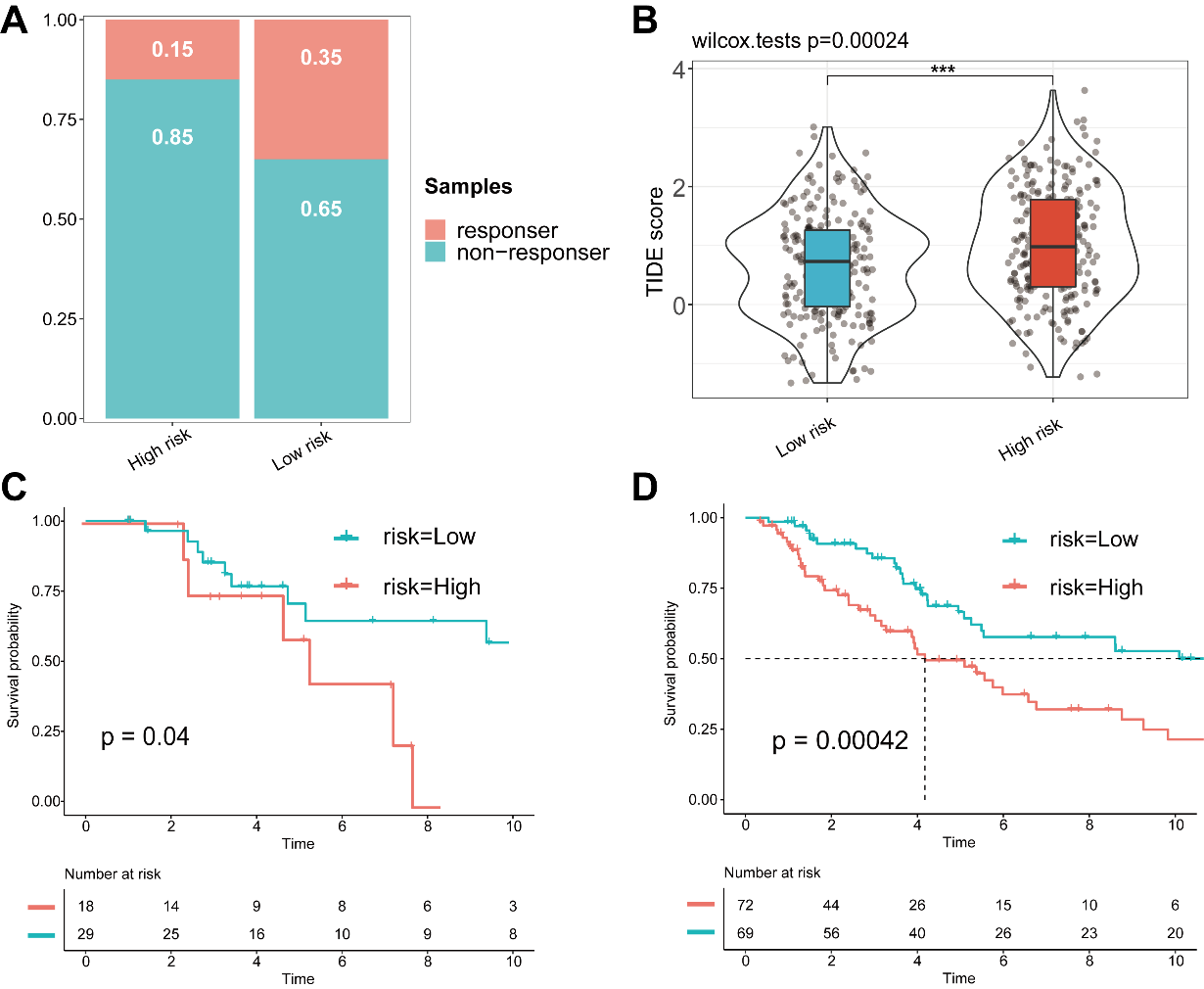


**Figure S9. Different responses to treatment between the two groups**. Based on the results of TIDE analysis, we compared the efficacy of immune checkpoint block and TIDE score in patients with low-risk and high-risk (**A and B**). The KM analysis of the patients who had received immunotherapy and chemotherapy between the two groups showed that the response of low-risk to immunotherapy and chemotherapy was significantly better than that of high-risk (**C and D**).

**
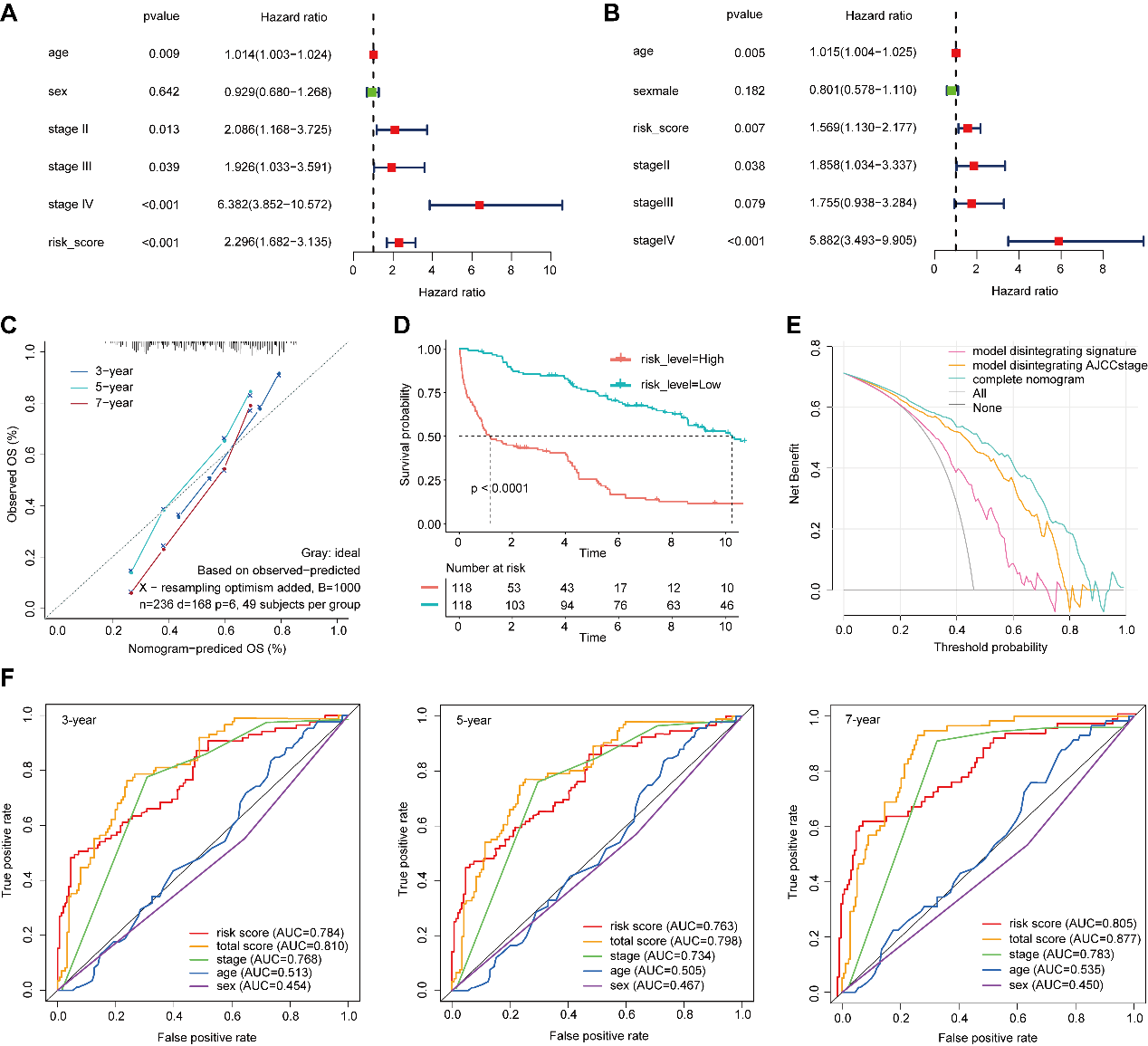
**

**Figure S10**. **Validation of the model in the validation cohort.** The univariate and multivariate Cox regression model (**A and B**). According to the calibration curve, predictive values were consistent with observed values considering the probabilities of 3-year, 5-year, and 7-year OS **(C**). Kaplan-Meier analysis showed that patients with a high-risk score had an obviously worse OS than patients with a low-risk score (**D**). Finally, clinical decision analysis (DCA) shows that the clinical benefit rate of the model without stage alone is higher than that of the model without ICBPS alone **(E**). The AUC values for 3-, 5-, and 7-year survival using the predictive nomogram reached 0.810, 0.798, and 0.877, respectively (**F**).


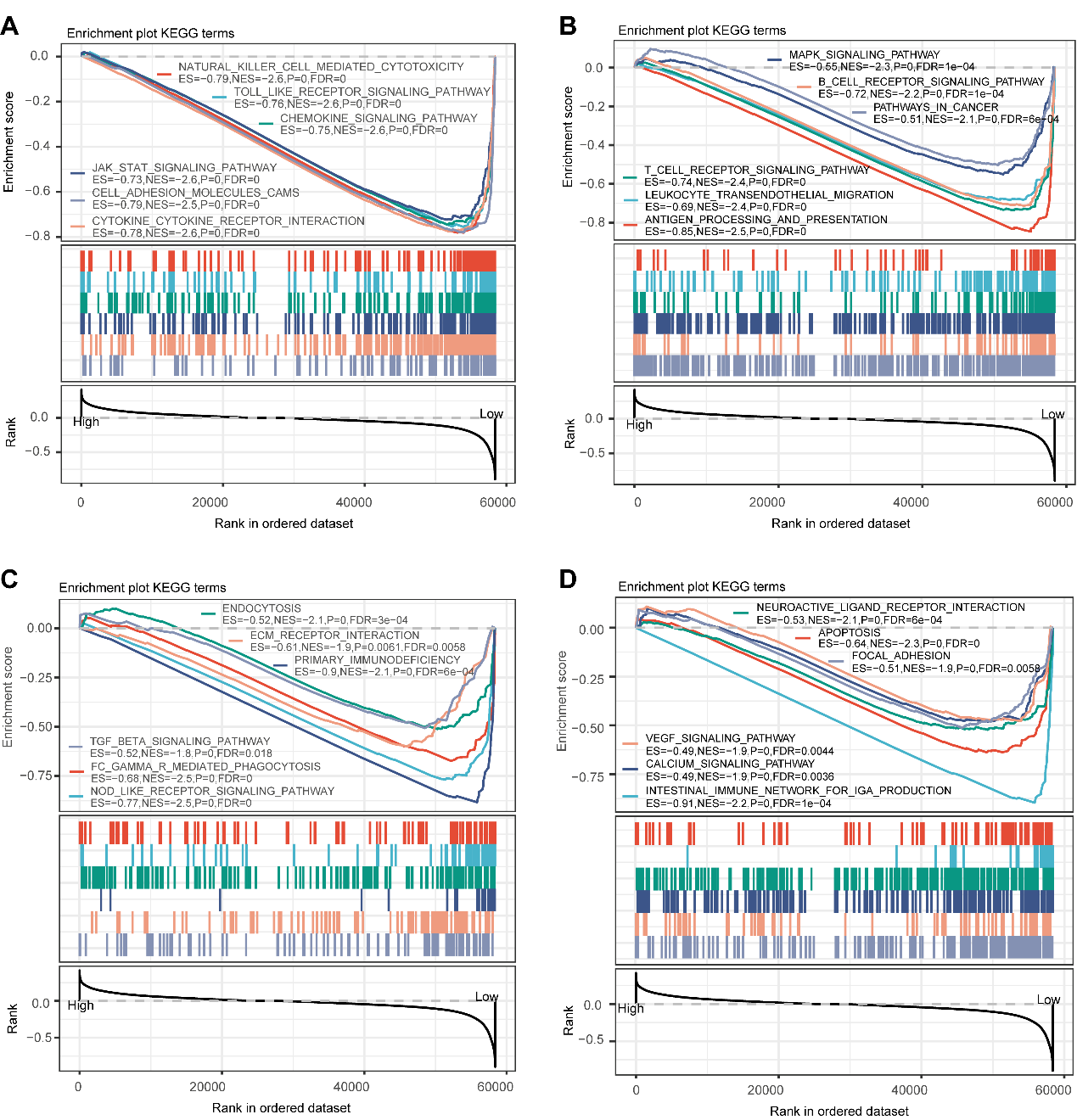


**Figure S11.** **GSEA analysis.** ES, enrichment score; NES, normalized enrichment score; P, P-value; FDR, adjusted P-value.
